# RES-Scanner: a software package for genome-wide identification of RNA-editing sites

**DOI:** 10.1186/s13742-016-0143-4

**Published:** 2016-08-18

**Authors:** Zongji Wang, Jinmin Lian, Qiye Li, Pei Zhang, Yang Zhou, Xiaoyu Zhan, Guojie Zhang

**Affiliations:** 1 School of Bioscience & Bioengineering, South China University of Technology, Guangzhou, 510006 China; 2 China National Genebank, BGI-Shenzhen, Shenzhen, 518083 China; 3 Centre for GeoGenetics, Natural History Museum of Denmark, University of Copenhagen, Øster Voldgade 5-7, 1350 Copenhagen K, Denmark; 4 College of Life Science and Technology, Jinan University, Guangzhou, 510000 China; 5 Centre for Social Evolution, Department of Biology, University of Copenhagen, Universitetsparken 15, DK-2100 Copenhagen, Denmark

**Keywords:** RES-Scanner, Software package, RNA editing, Genome-wide, Identification, Detection

## Abstract

**Background:**

High-throughput sequencing (HTS) provides a powerful solution for the genome-wide identification of RNA-editing sites. However, it remains a great challenge to distinguish RNA-editing sites from genetic variants and technical artifacts caused by sequencing or read-mapping errors.

**Results:**

Here we present RES-Scanner, a flexible and efficient software package that detects and annotates RNA-editing sites using matching RNA-seq and DNA-seq data from the same individuals or samples. RES-Scanner allows the use of both raw HTS reads and pre-aligned reads in BAM format as inputs. When inputs are HTS reads, RES-Scanner can invoke the BWA mapper to align reads to the reference genome automatically. To rigorously identify potential false positives resulting from genetic variants, we have equipped RES-Scanner with sophisticated statistical models to infer the reliability of homozygous genotypes called from DNA-seq data. These models are applicable to samples from either single individuals or a pool of multiple individuals if the ploidy information is known. In addition, RES-Scanner implements statistical tests to distinguish genuine RNA-editing sites from sequencing errors, and provides a series of sophisticated filtering options to remove false positives resulting from mapping errors. Finally, RES-Scanner can improve the completeness and accuracy of editing site identification when the data of multiple samples are available.

**Conclusion:**

RES-Scanner, as a software package written in the Perl programming language, provides a comprehensive solution that addresses read mapping, homozygous genotype calling, *de novo* RNA-editing site identification and annotation for any species with matching RNA-seq and DNA-seq data. The package is freely available.

**Electronic supplementary material:**

The online version of this article (doi:10.1186/s13742-016-0143-4) contains supplementary material, which is available to authorized users.

## Findings

### Introduction

RNA editing is a post-transcriptional-processing mechanism, which alters RNA sequences by insertion, deletion or modification of specific nucleotides so that the information in the mature RNA differs from that defined in the genome [1, 2]. In metazoa, the vast majority of RNA-editing events involve the deamination of adenosine (A) to inosine (I), which is catalyzed by a family of adenosine deaminases that act on RNA (ADARs) [2]. As inosine is recognized as guanosine (G) by other molecular machines in vivo, A-to-I editing provides a potential mechanism for diversifying the transcriptomes by recoding amino acids [2], changing messenger RNA (mRNA) splicing sites [3], editing microRNA (miRNA) sequences [4] or changing miRNA target sites in mRNA [5]. Other types of RNA-editing events (e.g. C-to-U, U-to-C and G-to-A editing) are also documented [6–9], but considered to be rare in metazoa. RNA editing appears to frequently target transcripts that encode proteins involved in fast neuronal signaling [10]. The dysregulation of RNA editing results in behavioral defects in *Caenorhabditis elegans* [11] and *Drosophila melanogaster* [12] and is associated with a variety of neurological diseases and cancers in humans [13, 14].

High-throughput RNA sequencing has enabled genome-wide identification of RNA-editing sites in any species. However, distinguishing RNA-editing events from genetic variants and technical artifacts caused by sequencing or read-mapping errors is still a challenge [15–18]. Much progress has been achieved in recent years in RNA-editing study at the genomic scale, resulting in the discovery of thousands to millions of RNA-editing sites in humans [19–24], rhesus macaques [25], mice [26], fruit flies [27], ants [28] and nematodes [29]. At the same time, several methodologies have been proposed to accurately identify RNA-editing sites with matching RNA-seq and DNA-seq data or with RNA-seq data alone [21–24]. However, published software packages devoted to this aim are scarce, especially for *de novo* identification of RNA-editing sites in non-model species.

REDItools was the first published software package for genome-wide RNA-editing site identification. It uses pre-aligned HTS reads in BAM format as inputs and implements a variety of filters to remove potential false positives [30]. However, it does not implement statistical models for determining homozygous genomic sites from DNA-seq data or for distinguishing real RNA-editing events from sequencing errors. GIREMI is a more recently released software package, which was developed to detect RNA-editing sites from RNA-seq data alone on the basis of allelic linkage and generalized linear models [24]. However, a comprehensive single-nucleotide polymorphism (SNP) dataset of the studied species must be available for GIREMI to estimate the reference mutual information (MI) distribution, which limits its application in non-model species that do not have such information. Thus far, an automatic tool that integrates read mapping, homozygous genotype calling, RNA-editing site identification and annotation for both model and non-model species is still lacking.

Here we introduce RES-Scanner (RES: RNA-editing site), a flexible and efficient software package written in the Perl programming language, which has been developed for genome-wide identification and annotation of RNA-editing sites. RES-Scanner is designed to address HTS read mapping, homozygous genotype calling, and *de novo* RNA-editing site identification and annotation for any species with matching RNA-seq and DNA-seq data.

### Methods and results

**Fig. 1:**
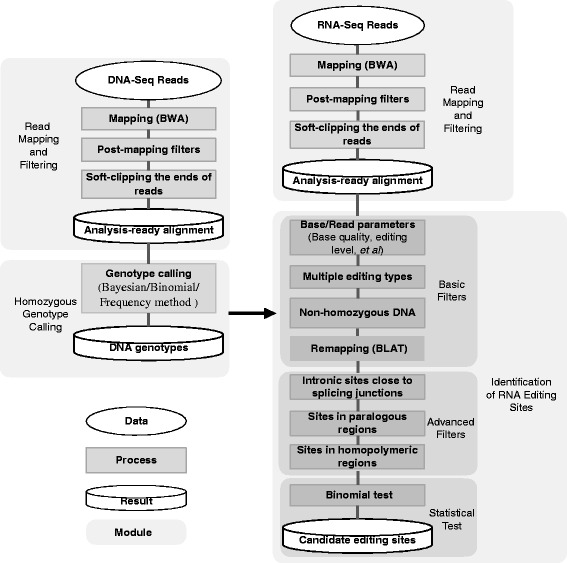
Overview of the workflow of RES-Scanner. RES-Scanner employs a three-part framework to detect RNA-editing sites with matching DNA-seq and RNA-seq data, including RNA/DNA-seq read mapping and filtering, homozygous genotype calling and identification of RNA-editing candidates

The prototype of RES-Scanner was developed during our previous study of RNA editomes in the leaf-cutting ant *Acromyrmex echinatior* [28]. To facilitate the use and portability of the processing pipeline, here we have developed it as a software package. RES-Scanner employs a three-part framework to detect RNA-editing sites, including RNA/DNA-seq read mapping and filtering, homozygous genotype calling and identification of RNA-editing candidates (Fig. 1). It takes Illumina HTS reads (single or paired-end) in FASTQ format as input, and supports RNA-seq data from both non-strand-specific and strand-specific libraries using the dUTP protocol [31].

#### RNA/DNA-seq read mapping and filtering

While the inputs for RES-Scanner are single/paired-end reads in FASTQ format, RES-Scanner can invoke BWA, an effective and accurate short-read aligner [32], to map the reads against a combination of a reference genome and exonic sequences surrounding all known splicing junctions (hereafter called junction sequences). Following Ramaswami et al. [22, 23], we set the length of the junction sequences to be slightly shorter than the read length to avoid simultaneous hits to the reference genome and the junction sequences. For example, when reads are 90 bp in length, a region of 89 bp upstream and downstream is selected. After BWA alignment, the locations of reads mapped on junction sequences are converted to regular genomic locations before subsequent analysis. We chose BWA as the default aligner in RES-Scanner, because the applicability and accuracy of BWA in genome-wide RNA-editing site detection have been acknowledged by many published studies for a series of species, including human [18, 22, 23, 33], macaque [25, 34], mouse [26], chicken [35], fly [23], *C. elegans* [29] and ant [28]. However, the default mapping strategy implemented in RES-Scanner cannot be optimal for every species, and so RES-Scanner can also accept as inputs pre-aligned DNA and RNA reads in BAM format from other aligners, such as Bowtie 2 [36], TopHat2 [37], GSNAP [38], and HISAT2 [39]. Thus, users are afforded considerable freedom in their choice of aligner to map the raw reads.

In the next step, RES-Scanner only keeps those reads with unique alignment and with no suboptimal hits using Perl scripts, and discards potential PCR duplications (i.e. read pairs that mapped to identical genomic locations) except for the one with the highest mapping quality according to the ‘rmdup’ function of SAMtools [40]. Given the higher error rate in Illumina sequencing at the ends of reads [41], the introduction of mismatches at the 5‘ read ends by random-hexamer priming during the first- and second-strand syntheses of RNA library construction [42], and the mapping errors at both the 5’ and 3’ ends resulting from the incorrect handling of insertions/deletions [43], false positives in RNA-editing sites are disproportionately increased at both ends of the reads. Thus, RES-Scanner clips the first and last few bases (six bases by default) of each aligned read before further analysis.

#### Homozygous genotype calling from DNA-seq data

Previous studies usually distinguished candidate RNA-editing events from genome-encoded variants based on frequency of the alternative allele (i.e. the allele that is not encoded in the reference genome) present in the DNA-seq data, followed by selection of an arbitrary threshold [19, 22, 25, 29]. However, this strategy may result in the under-estimation of heterozygous sites, especially when the sequencing depth is low or the sample is not diploid. Moreover, it does not provide a statistical measurement of uncertainty in the inference of homozygous genotypes.

RES-Scanner introduces a statistical model based on a Bayesian method (Bayesian model) to infer the genotype of each genomic site on the reference genome [44]. This statistical model can handle samples from one individual or a pool of multiple individuals based on the ploidy information supplied by the users (e.g. the ploidy for a sample from a diploid individual is two and that of a pooled sample with two diploid individuals is four). In brief, for a genomic site on the reference genome, our model computes the probabilities of all the possible genotypes based on the nucleotide bases, along with their sequencing quality scores, mapped to this genomic site, and then reports the genotype with the highest posterior probability. For example, for a sample from a diploid individual, all ten possible genotypes (i.e. AA, TT, CC, GG, AT, AC, AG, CT, CG and GT) are examined, while for a sample obtained by pooling two diploid individuals, all 22 possible genotypes are examined (for details see Additional file 1: Supplementary text 1). Finally, only the genomic sites reporting homozygous genotypes with posterior probabilities exceeding 0.95 or 0.99 and supported by a sufficient number of reads (i.e. ≥ 10×) are kept for RNA-editing site determination. In practice, RES-Scanner only applies the Bayesian procedure to candidate RNA-editing sites that satisfy some basic criteria (see below) in order to save computing time.

RES-Scanner also provides two additional methods to estimate the reliability of a homozygous genotype: one is based on a binomial distribution model (Binomial model) and the other is based on the read depth and the frequency of the alternative allele (Frequency model) present in the DNA-seq data (for details see Additional file 1: Supplementary texts 2 and 3).

#### Identification of RNA-editing sites

By default, RES-Scanner requires that a candidate RNA-editing site in a given sample must be supported by at least three non-redundant RNA reads that have been mapped on overlapping but not identical positions, and the editing level (i.e. the percentage of all reads mapping on that position that support editing) of this site must be ≥ 5 %. Bases on RNA reads with a Phred quality score below 30 are discarded, restricting the upper limit of sequencing error for an RNA base to < 0.1 % for any candidate editing site. Users can modify the quality score cutoff according to their dataset. These candidate editing sites are then subjected to genotype analysis as described above, and only the sites showing robust homozygosity in genomic DNA are kept. RES-Scanner also allows users to supply a list of potential SNPs derived from other analyses or databases of the target species, and exclude candidate editing sites that overlap these SNPs. Candidate sites that show multiple editing types are also discarded as these positions may be associated with higher probabilities of mapping error.

To avoid potential false positives resulting from misalignment of reads onto very similar paralogous regions, RES-Scanner invokes BLAT [45], a BLAST-like alignment tool with a fundamentally different algorithm from most short-read aligners, to realign all the reads that support RNA editing (i.e. reads showing a mismatch to the reference). Then, a read is defined as a qualifying read if (1) the best hit of this read overlaps its original candidate site and (2) the second-best hit, if it exists, has a BLAT score of < 95 % of the best hit. Only candidate editing sites for which the proportion of qualifying reads in relation to all BLAT-realigned reads exceeds 50 % are kept.

In addition to these basic filters, and to avoid potential false positives resulting from mis-mapping of reads at splice junctions, RES-Scanner requires a candidate editing site to be supported by at least one RNA read in the middle of its length (e.g. from positions 23 to 68 of a 90-bp read), and discards intronic candidate sites that occur within six bases of a splice site. RES-Scanner also removes sites in homopolymer runs of five or more base pairs (e.g. AAAAA), given that homopolymers have higher rates of sequencing error [41]. Finally, RES-Scanner also discards candidate editing sites with DNA read depths of more than twice the genome-wide peak or mean depth, as such sites are likely to be located in regions showing copy number variation (i.e. another form of highly similar paralogous region that is not fully present in the reference genome).

To further eliminate false positives due to sequencing errors, RES-Scanner performs statistical tests for all the candidate editing sites based on the binomial distribution B(k, n, p) [46], where p is set to be the upper limit of sequencing error for an RNA base as described above, n is equal to the total read depth of a given candidate site, and k denotes the number of reads supporting editing (for details see Additional file 1: Supplementary text 4). P-values are then adjusted by the Benjamini-Hochberg false discovery rate (FDR) [47], and only candidate sites with FDRs below a user-chosen cutoff (usually 0.01 or 0.05) are considered as true positives. Two examples from real data that show the positive contribution of binomial tests to reducing false positives resulting from sequencing error are shown in Additional file 1: Supplementary text 8.

#### Improvement of identification when multiple samples are available

Owing to the stringent filtering criteria of RES-Scanner in identifying RNA-editing sites for a single sample, a number of true positives may be missed due to their low editing level, insufficient sequencing depth or failure to satisfy other requirements. These editing sites can, however, be retrieved by RES-Scanner if multiple samples across individuals or tissues are available. RES-Scanner first combines all editing sites identified in each sample to obtain a comprehensive map of potentially editable positions in the genome of the target species. These positions are homozygous for DNA, located in unique genomic regions, not close to any splice sites and RNA-edited in at least one of the multiple samples. RES-Scanner then retrieves missed editing sites in each sample in these editable positions using the more liberal criteria of at least one RNA read supporting editing and an FDR below a user-chosen cutoff (usually 0.01 or 0.05). DNA data from multiple samples are also helpful in improving the accuracy of calling homozygous genomic sites. RES-Scanner will discard any editing site for which the genomic DNA is not homozygous in any one of the multiple DNA samples.

#### Annotation of RNA-editing sites

RES-Scanner can annotate the identified RNA-editing sites with a variety of genomic features, such as exon, intron, coding sequence (CDS), 5‘-untranslated region (5‘-UTR), 3‘-UTR and repeat, if the position files of these genomic features are provided (see Additional file 2 for format description). Moreover, for the editing sites targeting CDSs, RES-Scanner can further infer the codon and amino acid change after RNA editing.

#### The accuracy of RES-Scanner

To estimate the accuracy of RES-Scanner, we reanalyzed the leaf-cutting ant dataset from Li et al. (nine samples with matching DNA-seq and strand-specific RNA-seq data) [28] and the human GM12878 lymphoblastoid cell line dataset from the ENCODE project, which has been used as a benchmark by many studies (e.g. [20, 22, 24, 30]).

For the ant dataset, we ran RES-Scanner with default parameters, except for setting the ploidy to eight for the Bayesian model to determine homozygous genomic sites due to the special genetic background of the ant samples [28] (for details see Additional file 1: Supplementary text 5). We identified an average of 14,650 editing sites (range 10,282–20,234) per sample, with about 95 % representing A-to-I editing (Additional file 1: Table S1; Additional file 3). We then estimated the FDR using the TA-clonal sequencing data of 16 PCR amplicons generated by Li et al. [28]. In total, we found 76 editing sites (71 A-to-I sites and five non-A-to-I sites) distributed over these PCR amplicons, with only three of them (two A-to-I sites and one non-A-to-I site) failing to be confirmed due to the absence of an observable editing signal in TA-clonal sequencing data (Additional file 3), representing a FDR of 4 % (3/76). Furthermore, we observed that the editing levels calculated by RES-Scanner were consistent with the levels obtained by TA-clonal sequencing (Pearson‘s *r* = 0.90 and *p* < 10^−15^; Additional file 1: Figure S1).

We next tested the performance of the three models (Bayesian, Binomial and Frequency) on homozygous genotype calling and RNA-editing site identification based on the data of sample L363 from Li et al. [28]. In general, we found that the vast majority of homozygous genomic sites and RNA-editing sites identified were common to all three methods, indicating that they performed comparably well (Additional file 1: Figure S2). In practice, the Frequency model runs faster than the Binomial model, and the Binomial model faster than the Bayesian model. However, if the average depth of the DNA-seq data is either low (e.g. ≤ 10×) or particularly high (e.g. ≥ 50×), we recommend the use of the Bayesian or Binomial model to statistically estimate the genomic homozygosity for the candidate editing sites. This is expected to reduce false positives when DNA-seq depth is low and reduce false negatives when DNA-seq depth is high.

For the human dataset, we performed a comprehensive comparison of the results from RES-Scanner with those generated by Ramaswami et al. [22], REDItools [30] and GIREMI [24]. Briefly, we produced two versions of editing sites using RES-Scanner. The first version was derived from the pre-aligned GM12878 RNA reads in BAM format generated by Ramaswami et al. [22] (‘pre-aligned’ version), while the second version was derived from raw RNA reads of the GM12878 dataset (‘raw reads’ version). Matching DNA-seq data in BAM format were used in both versions (Additional file 1: Tables S2-S4). Following Ramaswami et al. [22] and Picardi et al. [30], we separated filtering criteria for RNA-editing candidates occurring in Alu repeats and non-Alu regions of the genome (for details see Additional file 1: Supplementary text 6). For the pre-aligned version, we identified 147,542 (A-to-I 96.36 %), 3,247 (A-to-I 97.04 %) and 1,163 (A-to-I 87.53 %) editing sites in Alu repeats, non-Alu repeats and nonrepetitive regions, respectively (Additional file 1: Table S5; Additional file 4). For the raw reads version, we obtained 149,710 (A-to-I 95.87 %), 2,794 (A-to-I 97.75 %) and 1,344 (A-to-I 81.32 %) editing sites in Alu repeats, non-Alu repeats and nonrepetitive regions, respectively (Additional file 1: Table S6; Additional file 5). These results were highly comparable with those reported in Ramaswami et al. [22] (Table 1). When compared with REDItools, RES-Scanner identified ~33 % fewer editing sites in Alu repeats (on average, 148,626 vs 221,401) with a higher proportion of A-to-I changes (96 vs 91 %), and ~29 % more sites in nonrepetitive regions (on average, 1,254 vs 887) with a lower proportion of A-to-I changes (84 vs 92 %). When compared with GIREMI, RES-Scanner identified approximately three times more editing sites in Alu repeats (148,626 vs 36,131), with a slightly lower proportion of A-to-I changes (96 vs 99 %), and about ten times more editing sites in non-Alu repeats (3,021 vs 267), with a much higher proportion of A-to-I changes (97 vs 84 %); the performance on nonrepetitive regions was comparable (Table 1). In addition, we observed that most editing sites identified by RES-Scanner were also common to the datasets of other studies (Additional file 1: Figure S3), and the proportions of non-synonymous and synonymous sites were also close to those in other studies (Additional file 1: Table S7). We further observed that editing sites detected by only one method tended to have a relatively low RNA read depth or an insufficient RNA editing signal, so that such candidate sites would be sensitive to the different mapping strategies and filtering processes adopted in different methods (for details see Additional file 1: Supplementary text 7).

**Table 1:** Performance of RES-Scanner compared with other methods applied to GM12878 human lymphoblastoid cell line data

	All		Alu		Repetitive non-Alu		Nonrepetitive	
	Total	% A-to-I	Total	% A-to-I	Total	% A-to-I	Total	% A-to-I
Ramaswami et al. [22]	150,865	95.7	147,029	95.8	2,385	97.4	1,451	86.6
REDItools [30]	222,288	91.3	221,401	91.2	Not investigated		887	92.2
GIREMI [24]	37,591	98.6	36,131	99.0	267	83.7	1,193	82.8
RES-Scanner (pre-aligned)	151,952	96.3	147,542	96.4	3,247	97.0	1,163	87.5
RES-Scanner (raw reads)	153,848	95.8	149,710	95.9	2,794	97.8	1,344	81.3

Taken together, these results indicate that RES-Scanner provides high accuracy in genome-wide RNA-editing site identification using matching DNA-seq and RNA-seq data, and it is capable of working on organisms with simple genomes like ants and complex genomes like humans.

#### The advantages of RES-Scanner relative to existing software

We first compared the overall runtime of RES-Scanner to REDItools and GIREMI in processing the human GM12878 dataset from pre-aligned reads to final editing sites. It is not surprising that GIREMI runs much faster than RES-Scanner (Table 2), as it only uses pre-aligned RNA reads in BAM format and a list of single-nucleotide variants (SNVs) as inputs [24] (for details see Additional file 1: Supplementary text 9). In other words, it does not need to process the DNA-seq data, which is usually much bigger than the RNA-seq data (e.g. in this case, ~150 Gb DNA-seq data vs ~9 Gb RNA-seq data; Additional file 1: Tables S2 and S3). However, the disadvantage of GIREMI is also obvious: it does not work for non-diploid samples or species with limited SNP information (e.g. most non-model species) [24]. REDItools is a software package that also uses matching DNA-seq and RNA-seq data for genome-wide identification of RNA-editing sites; as such, it should be more suitable for comparison. We used the pre-aligned DNA and RNA reads of the human GM12878 dataset in BAM format as inputs for both RES-Scanner and REDItools, and found that RES-Scanner only spent a third of the time taken by REDItools to obtain final editing sites from pre-aligned reads (Table 2; for details see Additional file 1: Supplementary text 10). It should be noted that RES-Scanner is designed to run multiple samples in parallel, so that an increase in sample numbers will not greatly affect the overall runtime if sufficient computing nodes are available. In fact, the limiting factor is usually the time required for the biggest chromosome, as each individual chromosome in a genome can also be run in parallel.

**Table 2:** Comparison of the cumulative CPU times (hours) for RES-Scanner, REDItools and GIREMI in processing the human GM12878 dataset from pre-aligned reads to final editing sites

Chromosome	RES-Scanner	REDItools	GIREMI
chr1	39.71	118.98	ND
chr2	44.51	128.62	ND
chr3	37.95	106.15	ND
chr4	29.83	81.61	ND
chr5	31.16	88.89	ND
chr6	28.99	85.49	ND
chr7	25.60	82.45	ND
chr8	25.38	64.02	ND
chr9	20.23	63.79	ND
chr10	23.01	71.45	ND
chr11	20.80	68.59	ND
chr12	23.33	75.51	ND
chr13	14.65	40.37	ND
chr14	16.10	51.55	ND
chr15	14.82	48.73	ND
chr16	15.64	48.15	ND
chr17	14.54	54.50	ND
chr18	13.03	35.29	ND
chr19	13.07	31.63	ND
chr20	12.19	31.04	ND
chr21	7.59	15.66	ND
chr22	7.67	21.62	ND
chrX	21.80	60.09	ND
chrY	1.62	1.94	ND
Total	503.23	1,476.12	10.87

Note: A total of 150 Gb of pre-aligned DNA reads and 9 Gb of pre-aligned RNA reads in BAM format were used as inputs for both RES-Scanner and REDItools, while 9 Gb of pre-aligned RNA reads and a list of SNVs derived from the RNA-seq data were used as inputs for GIREMI. The time for GIREMI included the cumulative CPU times of generating the SNV list from the RNA-seq data using SAMtools [40] (9.60 h) and running GIREMI (1.27 h). As GIREMI required all SNVs from the whole genome to construct the MI distribution, CPU times for individual chromosomes could not be determined. *ND* not determined

We also compared the performance of the three software packages (RES-Scanner, REDItools and GIREMI) on non-diploid samples from non-model species using the leaf-cutting ant dataset from Li et al. [28], in which each sample was a pool of multiple individuals from the same ant colony, representing samples with a ploidy of eight [28]. GIREMI is unable to detect RNA-editing sites in the ant samples because its MI model is specifically designed for diploids [24]. For REDItools and RES-Scanner, we used the same sets of DNA and RNA BAM files as inputs and used similar parameters for editing site identification (for details see Additional file 1:Supplementary text 10 and Table S8). However, for the parameter corresponding to the definition of homozygous genotypes from DNA-seq data in REDItools, we chose a series of cutoffs by limiting the maximal frequency of non-reference bases to be equal to 0, ≤ 0.02 and ≤ 0.05, respectively, as there was no prior knowledge about the optimal cutoff for samples with a ploidy of eight. We found that REDItools generally detected similar numbers of editing sites but with significantly lower A-to-I ratios when compared with RES-Scanner (~80 vs 94 %; Additional file 1: Table S8), implying that REDItools produced more false positives on the ant dataset given that non-A-to-I editing has been confirmed as rare in ants [28]. Although the performance of these software packages on other datasets besides the ant data remains to be investigated, it is noteworthy that RES-Scanner implements statistical models (Bayesian and Binomial) to infer the homozygous genotypes from DNA-seq data instead of choosing arbitrary thresholds. These models make RES-Scanner applicable for distinguishing RNA-editing sites from genetic variants for samples with any ploidy number, greatly enhancing the value of RES-Scanner for other datasets.

Finally, RES-Scanner can provide an integrated, end-to-end solution that works from raw sequencing reads to final editing sites, greatly reducing the risk of incompatibility between read alignment outputs and the downstream editing site identification pipeline. RES-Scanner can automatically annotate identified RNA-editing sites with genomic features and deduce codon and amino acid changes after RNA editing, providing useful information for downstream analysis. Furthermore, the final result file output by RES-Scanner has integrated DNA and RNA information from all samples for each editing site, greatly facilitating downstream comparative analysis between different samples. Thus, we believe that RES-Scanner is also superior to other existing software packages in terms of ease of use.

## Conclusions

Compared with existing packages, RES-Scanner provides four novel features:

1. It is equipped with rigorous statistical models (Bayesian and Binomial) to infer the reliability of homozygous genotypes derived from DNA-seq data. This approach is different from traditional SNP calling, which is aimed at ensuring the reliability of genetic polymorphism rather than homozygosity.
2. With these statistical models, RES-Scanner is capable of calling homozygous genotypes reliably from samples with any ploidy number, including samples from a pool of multiple individuals. This feature is especially useful because many species - or the target tissues of a species - are small in size and, in practice, pooling of multiple individuals is usually required to obtain enough biomass for sequencing.
3. RES-Scanner implements binomial tests to rigorously distinguish RNA-editing sites from sequencing errors by assigning a p-value to each RNA-editing candidate.
4. RES-Scanner provides a complete pipeline from raw sequencing reads to final editing sites, which should be especially valuable to users who have limited experience in bioinformatics or are working with non-model species with no prior knowledge of the optimal mapping strategy.

The application of our package to the leaf-cutting ant and GM12878 human datasets demonstrates that RES-Scanner provides high accuracy in identifying RNA-editing sites using matching DNA-seq and RNA-seq data. It should be noted that RES-Scanner is not species-specific: it is applicable to genome-wide identification of RNA-editing sites in any species with matching RNA-seq and DNA-seq data.

A detailed user manual for RES-Scanner is available in Additional file 2.

### Availability and requirements

- **Project name**: RES-Scanner
- **Project home page**: https://github.com/ZhangLabSZ/RES-Scanner
- **Operating systems**: Linux/Mac OS X
- **Programming language**: Perl
- **Requirements**: See Additional file 2 for a comprehensive list of dependencies
- **License**: GPL v3
- **Restrictions to use by non-academics**: None

## Abbreviations

FDR, false discovery rate; HTS, high-throughput sequencing; MI, mutual information; SNP, single-nucleotide polymorphism; SNV, single-nucleotide variant

## Additional files

Additional file 1: Supplementary texts, figures and tables. (DOCX 527 kb) Additional file 2: User manual for RES-Scanner. (DOCX 70 kb) Additional file 3: RNA-editing sites of the leaf-cutting ant identified by RES-Scanner. (XLSX 4.44 mb) Additional file 4: RNA-editing sites of the GM12878 human cell line (‘pre-aligned’ version). Identified by RES-Scanner using pre-aligned RNA reads in BAM format generated by Ramaswami et al. [22] as inputs. (XLSX 19036 kb) Additional file 5: RNA-editing sites of the GM12878 human cell line (‘raw reads’ version). Identified by RES-Scanner using raw RNA reads as inputs. (XLSX 19040 kb)
