# Supplementary material for: RES-Scanner: a software package for genome-wide identification of RNA-editing sites
Source: Gigascience. 2016 Aug 18;5:37. doi: 10.1186/s13742-016-0143-4 (PMC4989487; doi:10.1186/s13742-016-0143-4)
Supplement: Additional file 1: — Supplementary texts, figures and tables. (DOCX 527 kb) [file 13742_2016_143_MOESM1_ESM.docx]

**Supplementary Texts**

**1. The *Bayesian* model for calling homozygous genomic sites from DNA-seq data**

Our Bayesian core computes the probabilities of all the possible genotypes using the observed data (i.e. the mapped bases and corresponding sequencing qualities) at a locus on the reference genome, and the genotype with the highest probability is reported. For a sample of a diploid individual, all ten possible genotypes (i.e. AA, TT, CC, GG, AT, AC, AG, CT, CG and GT) will be tested. According to a previous study on human population [[1](#_ENREF_1)], only ~0.2% of SNPs are triallelic, indicating that multiallelic (i.e. triallelic or tetrallelic) genotypes are rare. Furthermore, multiallelic genotypes do not have an impact on our homozygous genotype calling based on our evaluation, as even heterozygous biallelic genotypes will have higher Bayesian posterior probabilities than homozygous genotypes if this locus is actually triallelic or tetrallelic. Thus, for a sample pooled from multiple individuals, we also assume a maximum of two alleles occurring at a locus. For instance, for a sample pooled from two diploid individuals, there are a total of 22 possible genotypes that will be tested, namely four homozygous tetraploid genotypes (AAAA, TTTT, CCCC and GGGG) plus 18 heterozygous tetraploid genotypes (e.g. ATTT, AATT and AAAT for a locus with A and T alleles).

Under a Bayesian model, the probability of the genotype G based on the observed data D (i.e. the mapped bases and corresponding sequencing qualities) at a particular locus in a sample can be expressed as:

$$p(G|D)=\frac{p\left( G \right)p(D|G)}{p(D)}$$

For each locus, the prior probability p(G) of each genotype is calculated according to the homozygosity level of the genome, the ratio of transition (i.e. changes between T and C or between A and G) over transversion (all other changes), and the GC content of the genome. The homozygosity level of a genome denotes the prior probability of a genomic site being homozygous, and can be easily obtained by counting the homozygous and heterozygous genomic sites in an SNP-calling analysis using the DNA-seq data, followed by calculating the ratio of homozygous sites over all the called genomic sites. In general, and based on our previous surveys in a wide range of species, the homozygosity level for a sample of a diploid individual is seldom lower than 0.99. For nuclear genes, the transition/transversion ratio may be slightly different among species and even among different parts of a genome [[2](#_ENREF_2)], and the typical estimations of the transition/transversion ratio range from 1.5 to 5 [[3](#_ENREF_3)]. Here we recommend a value of 2 as the default transition/transversion ratio across the entire genome. However, users are allowed to specify their own transition/transversion ratios with a parameter incorporated in RES-Scanner. For example, for a diploid genome with a homozygosity level of 0.99, a transition/transversion ratio of 2 and a GC content of 0.336, the prior probabilities of the homozygous diploid genotypes p(CC) and p(GG) are both calculated as 0.99×0.336/2 = 0.16632, while p(AA) and p(TT) are both calculated as 0.99×(1-0.336)/2 = 0.32868 (i.e. homozygous ratio times each base content). For the heterozygous genotypes, p(AG) and p(TC) are both calculated as (1-0.99)×2/((2×2)+4) = 0.00250, while p(AT), p(AC), p(TG) and p(CG) are calculated as (1-0.99)×1/((2×2)+4) = 0.00125 (i.e. heterozygous ratio divided by the proportion of heterozygous genotypes).

The value p(D) can be calculated as the sum of a weighted average of the conditional probabilities of D given each genotype, which is constant over all genotypes when we adopt the total probability rule [[4](#_ENREF_4)]. The likelihood p(D|G) for the assumed genotype is calculated from each base b covering the target locus (i.e. the *pileup* of bases), which can be formulated as:

$$p\left( D | G \right)=\prod_{b\in\mathrm{pileup}} p(b|G)$$

Supposing that the n sets (n is the ploidy level) of chromosomes in a genome are independent, the probability of each base given the genotype is defined as:

$$p\left( b | G \right)= p\left( b | \left\{ A1,A2 \right\} \right)=\frac{m}{n}p\left( b | A1 \right)+ \frac{n-m}{n}p(b|A2)$$

where the genotype is decomposed into its two alleles (A1 and A2), and m is the copy number of allele A1 in a given ploidy.

Because the Illumina sequencer uses two lasers to distinguish the four nucleotides, with a green laser used to detect G and T at the same time, and a red laser to detect A and C, the highest base substitution errors observed are between A/C and G/T [[5](#_ENREF_5),[6](#_ENREF_6)]. To compensate for the unequal substitution errors between A/C or G/T and other substitutions, we used a weighting scheme to recalibrate the quality scores (defaulted by approximately setting the weight of A/C and G/T to 0.5 and the weight of the other substitutions to 0.25 based on previous estimation) [[7](#_ENREF_7)]. The probability of a given allele is:

$$p\left( b | A \right)=\left\{ \begin{aligned} e*s :b\neq A \\ 1-e :b=A \end{aligned} \right.$$

where e is the reversed Phred-scaled quality score at the base.

**2. The *Binomial* model for calling homozygous genomic sites from DNA-seq data**

Under the *Binomial* model, RES-Scanner performs a binomial test to estimate the heterozygosity of genomic sites corresponding to all of the candidate editing sites based on the formula:

$$P\mathrm{value}=\sum_{m=0}^{k} \binom{n}{m}p^{m}q^{n-m} , m\leq n$$

where p is the reciprocal of the ploidy level provided by users, namely the lowest allele frequency that could be observed under a given ploidy level. For example, for a sample of a diploid individual, the ploidy level is two and p is set to ½. For a sample pooled from two diploid individuals, the ploidy level is four and p is set to ¼. Parameter n represents the total number of DNA reads covering a given genomic site after excluding the reads with Phred base quality scores below the user-provided cutoff for that site. Parameter k denotes the number of reads reporting an alternative allele (i.e. the allele that is not encoded in the reference genome) for that site. The P-value of this binomial test represents the probability of observing ≤ k reads reporting an alternative allele under the lowest allele frequency (p) and the total number of reads covering a given genomic site (n). An extremely small P-value suggests that it is unlikely that as few as k reads reporting an alternative allele under p and n will be observed, thus indicating that the genomic site is a homozygous one. In practice, all the non-reference-encoded alleles observed from the DNA reads at a given genomic site are treated as the alternative allele. P-values are further adjusted by the Benjamini-Hochberg false discovery rate (FDR) [[8](#_ENREF_8)], and sites with FDRs below a user-chosen cutoff (usually 0.01 or 0.05) are considered as reliable homozygous genomic sites.

**3. The *Frequency* model for calling homozygous genomic sites from DNA-seq data**

Under the *Frequency* model, RES-Scanner will simply determine a homozygous genomic site by counting the number of DNA reads covering that site and calculating the frequency of the alternative allele present in the DNA reads. All the non-reference-encoded alleles observed from the DNA reads for a given genomic site are treated as the alternative allele. Thus, this model will run particularly quickly. In general, RES-Scanner requires a DNA read depth ≥ 10X and frequency ≤ 0.1 for a sample of a diploid individual, and more stringent thresholds are recommended for samples with higher ploidy levels. In practice, the *Frequency* model runs faster than the *Binomial* model, and the *Binomial* model runs faster than the *Bayesian* model. However, if the average depth of the DNA-seq data is low (e.g. ≤ 10X) or particularly high (e.g. ≥ 50X), we recommend selection of the *Bayesian* or *Binomial* model to statistically estimate the genomic homozygosity for the candidate editing sites. This is expected to reduce false positives when DNA-seq depth is low and reduce false negatives when DNA-seq depth is high.

**4. The *Binomial* model for distinguishing RNA-editing sites from sequencing errors**

To estimate the probability that the observed editing sites have resulted from sequencing errors, RES-Scanner performs a binomial test based on the formula:

$$P\mathrm{value}=\sum_{m=k}^{n} \binom{n}{m}p^{m}q^{n-m}, m\leq n$$

where p is the upper limit of sequencing error for an RNA base according to the user-provided Phred base quality score cutoff. For example, if the base quality score cutoff is set to 30, the upper limit of sequencing error p is 0.1%. Parameter n represents the total number of RNA reads covering a given genomic site after excluding those reads with Phred base quality scores below the user-provided cutoff for that site. Parameter k denotes the number of reads supporting RNA editing at that site. The P-value of this binomial test represents the probability of observing ≥ k reads supporting editing under the upper limit of sequencing error (p) and the total number of reads covering a given genomic site (n). An extremely small P-value suggests that it is unlikely that as many as k reads supporting editing under p and n will be observed. P-values are further adjusted by FDR [[8](#_ENREF_8)], and sites with FDRs below a user-chosen cutoff (usually 0.01 or 0.05) are considered as bona fide RNA-editing sites.

**5. Analysis of leaf-cutting ant data with RES-Scanner**

RES-Scanner has been tested on nine leaf-cutting ant samples (three female castes for each of three colonies), from which deep DNA-seq (~39X/sample) and strand-specific RNA-seq data (~37X/sample) were generated in our previous study of the RNA editomes of *Acromyrmex echinatior* [[9](#_ENREF_9)]. The DNA-seq and RNA-seq data of *A. echinatior* can be downloaded from the NCBI Gene Expression Omnibus (GEO) [14] under accession number GSE51576, and the reference genome (Aech_2.0_scaffolds.fa.gz) and gene annotation (v3.8) of *A. echinatior* are also available to download [15].

First, we manually created the configuration file RES-Scanner_alignment_config.txt for the RES-Scanner alignment pipeline according to the user manual (see Additional file 5). Then we aligned the DNA-seq and RNA-seq reads to the reference genome using the following RES-Scanner alignment command lines (assuming a working directory of /usr/bin/ant/):

perl RES-Scanner_alignment.part1.pl --outDir /usr/bin/ant/alignment/ --ref Aech_v2.0.fa --bwa /usr/bin/bwa-0.5.9/bwa --index 1 --config /usr/bin/ant/RES-Scanner_alignment_config.txt --split --junction /usr/bin/ant/annotation/splicesites.pos

perl RES-Scanner_alignment.part2.pl --ref Aech_v2.0.fa --config /usr/bin/ant/RES-Scanner_alignment_config.txt --outDir /usr/bin/ant/alignment/ --bwa /usr/bin/bwa-0.5.9/bwa --samtools /usr/bin/samtools-0.1.18/samtools --index 1 -n 9 --t 4

As this RES-Scanner alignment step completes, the configuration file for the RES-Scanner identification step is automatically generated in directory /usr/bin/ant/alignment/*.* It is notable that we set --ploidy to 8 for the *Bayesian* model to determine homozygous genotypes in the identification step, because each of the nine samples were in fact pooled from 20 to 50 individuals, while *A. echinatior*, similarly to most other eusocial Hymenoptera, has low degrees of genetic polymorphism within single colonies as all females are offspring of the same diploid queen who mated with approximately six haploid males [[10](#_ENREF_10)]. The command line for the RES-Scanner identification step was:

perl RES-Scanner_identification.pl --config /usr/bin/ant/alignment/RES_Scanner_indentification_config.txt --out /usr/bin/ant/RES_Scanner_indentification_outdir/ --genome Aech_v2.0.fa -ss 1 --samtools /usr/bin/samtools-0.1.18/samtools --blat /usr/bin/blat --trim 6,6 --q 30 --mq 20 --ploidy 8 --rmdup 1 --junctionCoordinate /usr/bin/ant/alignment/index/junctionFlankSequenceRegion.txt --paralogous_R 1 --paralogous_D 1 --homopolymer 1 --posdir /usr/bin/ant/posdir/

Finally, the RNA-editing sites of the nine samples, complete with annotation, are generated in the file /usr/bin/ant/RES_Scanner_indentification_outdir/RES_final_result.annotation.

**6. Analysis of human data with RES-Scanner**

RES-Scanner has also been tested on the human GM12878 lymphoblastoid cell line dataset, which also has deep DNA-seq and strand-specific RNA-seq data [[11](#_ENREF_11),[12](#_ENREF_12)]. Specifically, we conducted two versions of editing site identification using RES-Scanner. The first version was started from pre-aligned DNA and RNA reads in BAM format ('pre-aligned' version), and the second version was started from raw RNA reads in FASTQ format and pre-aligned DNA reads in BAM format ('raw reads' version).

Pre-aligned DNA-seq reads in BAM format for the GM12878 cell line were downloaded from the FTP server of the 1000 Genomes Project [16] and, before being input to RES-Scanner, the DNA BAM files were re-headered using SAMtools' reheader. Specifically, the following files were downloaded:

- NA12878.chrom1.ILLUMINA.bwa.CEU.high_coverage.20100311.bam
- NA12878.chrom10.ILLUMINA.bwa.CEU.high_coverage.20100311.bam
- NA12878.chrom11.ILLUMINA.bwa.CEU.high_coverage.20100311.bam
- NA12878.chrom12.ILLUMINA.bwa.CEU.high_coverage.20100311.bam
- NA12878.chrom13.ILLUMINA.bwa.CEU.high_coverage.20100311.bam
- NA12878.chrom14.ILLUMINA.bwa.CEU.high_coverage.20100311.bam
- NA12878.chrom15.ILLUMINA.bwa.CEU.high_coverage.20100311.bam
- NA12878.chrom16.ILLUMINA.bwa.CEU.high_coverage.20100311.bam
- NA12878.chrom17.ILLUMINA.bwa.CEU.high_coverage.20100311.bam
- NA12878.chrom18.ILLUMINA.bwa.CEU.high_coverage.20100311.bam
- NA12878.chrom19.ILLUMINA.bwa.CEU.high_coverage.20100311.bam
- NA12878.chrom2.ILLUMINA.bwa.CEU.high_coverage.20100311.bam
- NA12878.chrom20.ILLUMINA.bwa.CEU.high_coverage.20100311.bam
- NA12878.chrom21.ILLUMINA.bwa.CEU.high_coverage.20100311.bam
- NA12878.chrom22.ILLUMINA.bwa.CEU.high_coverage.20100311.bam
- NA12878.chrom3.ILLUMINA.bwa.CEU.high_coverage.20100311.bam
- NA12878.chrom4.ILLUMINA.bwa.CEU.high_coverage.20100311.bam
- NA12878.chrom5.ILLUMINA.bwa.CEU.high_coverage.20100311.bam
- NA12878.chrom6.ILLUMINA.bwa.CEU.high_coverage.20100311.bam
- NA12878.chrom7.ILLUMINA.bwa.CEU.high_coverage.20100311.bam
- NA12878.chrom8.ILLUMINA.bwa.CEU.high_coverage.20100311.bam
- NA12878.chrom9.ILLUMINA.bwa.CEU.high_coverage.20100311.bam
- NA12878.chromMT.ILLUMINA.bwa.CEU.high_coverage.20100311.bam
- NA12878.chromX.ILLUMINA.bwa.CEU.high_coverage.20100311.bam
- NA12878.chromY.ILLUMINA.bwa.CEU.high_coverage.20100311.bam

For the pre-aligned version, Ernesto Picardi from the University of Bari kindly provided us with the BAM files (after removing PCR duplicates) of pre-aligned RNA-seq reads that were originally generated by Ramaswami *et al.* using BWA [[12](#_ENREF_12)]. Although the RNA reads were sequenced from a paired-end protocol, Ramaswami *et al.* mapped each end of a read pair independently onto a combination of the hg19 reference genome and exonic sequences surrounding all known splicing junctions. The pre-aligned RNA reads were presented as four BAM files:

- LID16629.r1.rmdup.bam # replicate 1, forward reads
- LID16629.r2.rmdup.bam # replicate 1, reverse reads
- LID16630.r1.rmdup.bam # replicate 2, forward reads
- LID16630.r2.rmdup.bam # replicate 2, reverse reads

Before running RES-Scanner, we adjusted the FLAG values of the RNA BAM files so that the forward reads and the reverse reads could be automatically distinguished by RES-Scanner, and modified the quality scores of reads from a base of ASCII-64 to ASCII-33. We then merged the four BAM files into one, re-sorted the contents and then divided the BAM file according to chromosome.

We also prepared POS files for genic features, including exon, intron and coding sequence, based on the gene annotation GTF file downloaded from Ensembl [17] (release-75) and POS files for repeats based on annotations (rmsk.txt.gz, simpleRepeat.txt.gz, chromOut.tar.gz and chromTrf.tar.gz) from [18]. *Alu* annotation was retrieved from the file chromOut.tar.gz and rmsk.txt.gz. Simple repeat annotation was processed from the file simpleRepeat.txt.gz directly. All known human SNPs were downloaded from UCSC [19], and the SNP list was converted to GFF3 format before input to RES-Scanner.

As inputs were pre-aligned reads, we only needed to run the RES-Scanner identification pipeline. The 25 chromosomes (22 + X + Y + MT) of the human genome were run in parallel. The configuration file for RES-Scanner, using chr21.config.txt as an example, was written as follows:

chr21 /usr/bin/hg19/DNA_bam/chr21_DNA.bam /usr/bin/hg19/RNA_bam/chr21_RNA.bam

Following Ramaswami *et al*. [[12](#_ENREF_12)] and Picardi *et al*. [[13](#_ENREF_13)], we separated filtering criteria for RNA-editing candidates occurring in *Alu* repeats and non-*Alu* regions of the genome. We also followed the criteria used in Ramaswami *et al*. when running RES-Scanner. Thus, to identify editing sites in *Alu* regions, the following command line was used:

perl /usr/bin/RES-Scanner/RES-Scanner_identification/RES-Scanner_identification.pl --config /usr/bin/hg19/chr21.config.txt --out /usr/bin/hg19/Alu/ --genome /usr/bin/hg19/hg19.fa --ss 1 --samtools /usr/bin/samtools-0.1.18/samtools --trim 6,0 --q 25 --mq 20 --DNAdepth 10 --RNAdepth 2 --editLevel 0 --editDepth 2 --refined 0 --paralogous_R 0 --paralogous_D 0 --homopolymer 0 --posdir /usr/bin/annotation/hg19/posdir/ --intronic 0 --editPvalue 1 --rmdup 0 --knownSNP /usr/bin/hg19/annotation/databaseSNP/snp142.txt.gz --uniqTag 0 --run

Then the editing sites in *Alu* repeats were recovered via the command line:

grep ‘Alu’ /usr/bin/hg19/Alu/RES_final_result.annotation > /usr/bin/hg19/Alu/RES_final_result.annotation.Alu

For editing sites in non-*Alu* regions, the command line for RES-Scanner was:

perl /usr/bin/RES-Scanner/RES-Scanner_identification/RES-Scanner_identification.pl --config /usr/bin/hg19/chr21.config.txt --out /usr/bin/hg19/nonAlu/ --genome /usr/bin/hg19/hg19.fa --ss 1 --samtools /usr/bin/samtools-0.1.18/samtools --blat /usr/bin/blat_64/blat --trim 6,0 --q 25 --mq 20 --DNAdepth 10 --RNAdepth 3 --editLevel 0.1 --editDepth 3 –refined 1 --refinedDepth 1 –readType 3 --paralogous_R 1 --paralogous_D 1 --homopolymer 1 --posdir /usr/bin/annotation/hg19/posdir/ --intronic 4 --editPvalue 0.05 --rmdup 0 --bestHitRatio 0.6 --knownSNP /usr/bin/hg19/annotation/databaseSNP/snp142.txt.gz --uniqTag 0 --run

Then editing sites in non-*Alu* repeat regions were recovered via:

grep –v ‘Alu’ /usr/bin/hg19/nonAlu/RES_final_result.annotation | grep ‘repeat’ > /usr/bin/hg19/nonAlu/RES_final_result.annotation.nonAluRepeat

Finally, editing sites in non-repeat regions were recovered via:

grep –v ‘repeat’ /usr/bin/hg19/nonAlu/RES_final_result.annotation > /usr/bin/hg19/nonAlu/RES_final_result.annotation.nonRepeat

For the raw reads version, we downloaded the raw RNA reads for GM12878 from UCSC [20]. The following four files were retrieved:

- wgEncodeCshlLongRnaSeqGm12878CellPapFastqRd1Rep1.fastq.gz
- wgEncodeCshlLongRnaSeqGm12878CellPapFastqRd1Rep2.fastq.gz
- wgEncodeCshlLongRnaSeqGm12878CellPapFastqRd2Rep1.fastq.gz
- wgEncodeCshlLongRnaSeqGm12878CellPapFastqRd2Rep2.fastq.gz

To conduct a paired-end alignment with the RES-Scanner alignment pipeline, a configuration file RES-Scanner_alignment_config.txt was created manually as below:

RNA Gm12878 wgEncodeCshlLongRnaSeqGm12878CellPapRep1 50000 /usr/bin/hg19/RNA-seq/wgEncodeCshlLongRnaSeqGm12878CellPapFastqRd1Rep1.fastq.gz /usr/bin/hg19/RNA-seq/wgEncodeCshlLongRnaSeqGm12878CellPapFastqRd2Rep1.fastq.gz

RNA Gm12878 wgEncodeCshlLongRnaSeqGm12878CellPapRep2 50000 /usr/bin/hg19/RNA-seq/wgEncodeCshlLongRnaSeqGm12878CellPapFastqRd1Rep2.fastq.gz /usr/bin/hg19/RNA-seq/wgEncodeCshlLongRnaSeqGm12878CellPapFastqRd2Rep2.fastq.gz

Then the following command lines were run:

perl /usr/bin/RES-Scanner/RES-Scanner_alignment/RES-Scanner_alignment.part1.pl --outDir /usr/bin/hg19/RNA_bam/ --ref /usr/bin/hg19/hg19.fa --bwa /usr/bin/bwa-0.5.9/bwa --index 1 --config /usr/bin/hg19/RNA_bam/RES-Scanner_alignment_config.txt --junction /usr/bin/hg19/splicesites_hg19.pos --split

perl /usr/bin/RES-Scanner/RES-Scanner_alignment/RES-Scanner_alignment.part2.pl --ref /usr/bin/hg19/hg19.fa --config /usr/bin/hg19/RNA_bam/RES-Scanner_alignment_config.txt --outDir /usr/bin/hg19/RNA_bam/ --bwa /usr/bin/bwa-0.5.9/bwa --samtools /usr/bin/samtools-0.1.18/samtools --index 1 --n 0.04 --t 4

After the RES-Scanner alignment pipeline had finished, the final BAM file was present in the directory /usr/bin/hg19/RNA_bam/RNA/Gm12878/. The BAM file was then divided according to chromosome, so that the 25 chromosomes (22 + X + Y + MT) of the human genome could be run in parallel. The configuration file for RES-Scanner identification, using chr21.config.txt as an example, was written as follows:

chr21 /usr/bin/hg19/DNA_bam/chr21_DNA.bam /usr/bin/hg19/RNA_bam/chr21_RNA.bam

To identify editing sites in *Alu* regions, the following command line was used:

perl /usr/bin/RES-Scanner/RES-Scanner_identification/RES-Scanner_identification.pl --config /usr/bin/hg19/chr21.config.txt --out /usr/bin/hg19/Alu/ --genome /usr/bin/hg19/hg19.fa -ss 1 --samtools /usr/bin/samtools-0.1.18/samtools --trim 6,0 --q 25 --mq 20 --DNAdepth 10 --RNAdepth 2 --editLevel 0 --editDepth 2 --refined 0 --paralogous_R 0 --paralogous_D 0 --homopolymer 0 --posdir /usr/bin/annotation/hg19/posdir/ --intronic 0 --editPvalue 1 --rmdup 0 --knownSNP /usr/bin/hg19/annotation/databaseSNP/snp142.txt.gz --uniqTag 0 --run

Then the editing sites in *Alu* repeats were recovered via the command line:

grep ‘Alu’ /usr/bin/hg19/Alu/RES_final_result.annotation > /usr/bin/hg19/Alu/RES_final_result.annotation.Alu

To identify editing sites in non-*Alu* regions, the command line used was:

perl /usr/bin/RES-Scanner/RES-Scanner_identification/RES-Scanner_identification.pl --config /usr/bin/hg19/chr21.config.txt --out /usr/bin/hg19/nonAlu/ --genome /usr/bin/hg19/19.fa --ss 1 --samtools /usr/bin/samtools-0.1.18/samtools --blat /usr/bin/blat_64/blat --trim 6,0 --q 25 --mq 20 --DNAdepth 10 --RNAdepth 3 --editLevel 0.1 --editDepth 3 --refined 1 --refinedDepth 1 --readType 3 --paralogous_R 1 --paralogous_D 1 --homopolymer 1 --posdir /usr/bin/annotation/hg19/posdir/ --intronic 4 --editPvalue 0.05 --rmdup 1 --bestHitRatio 0.6 --knownSNP /usr/bin/hg19/annotation/databaseSNP/snp142.txt.gz --uniqTag 0 --run

Then editing sites in non-*Alu* repeat regions were recovered via:

grep –v ‘Alu’ /usr/bin/hg19/nonAlu/RES_final_result.annotation | grep ‘repeat’ > /usr/bin/hg19/nonAlu/RES_final_result.annotation.nonAluRepeat

Finally, editing sites in non-repeat regions were recovered via:

grep –v ‘repeat’ /usr/bin/hg19/nonAlu/RES_final_result.annotation > /usr/bin/hg19/nonAlu/RES_final_result.annotation.nonRepeat

For comparative analysis, the RNA-editing sites of Ramaswami *et al.* [[12](#_ENREF_12)] were retrieved from Supplementary Data 1 of their paper, and the editing sites identified in the original paper of REDItools [[13](#_ENREF_13)] were kindly provided by Dr. Ernesto Picardi of the University of Bari.

**7. Potential reasons for the imperfect overlap of editing sites between methods**

To investigate the potential reasons for the imperfect overlap of editing sites identified by different methods (see Figure S3), we divided RNA-editing sites from Ramaswami *et al.* [[12](#_ENREF_12)], REDItools [[13](#_ENREF_13)] and RES-Scanner (raw reads version) into two parts: (1) overlapped sites that are shared by at least one of the other two methods; (2) method-specific sites. Then we compared the RNA/DNA read depths and editing levels between these two parts. We found that the RNA read depths of the overlapped sites were consistently and significantly higher than that of the method-specific sites (Wilcoxon rank-sum test p < 10^-15^; Figure S4A). A similar pattern was also observed in the comparison of editing levels (Wilcoxon rank-sum test p < 10^-15^; Figure S4C), but not in the comparison of DNA read depths (Figure S4B). Specifically, we observed that the majority of method-specific sites from REDItools and Ramaswami *et al.* (98.36% and 87%, respectively) had ≤ 2 RNA reads supporting RNA editing in the RES-Scanner dataset. These results indicate that editing sites detected by only one method tend to have a relatively low RNA read depth or an insufficient RNA editing signal, making such candidate sites sensitive to the different mapping strategies and filtering processes adopted by different methods, and providing an explanation for the imperfect overlap between methods.

**8. The contribution of binomial tests to reducing false positives due to sequencing error**

The contribution of binomial tests to reducing false positives can be observed in our analysis of the human and ant datasets. For example, during the identification of editing sites in *Alu* regions of the human dataset, we obtained 159,149 (7% non-A-to-I) and 149,710 (4% non-A-to-I) editing sites before and after binomial test filtering, respectively. For the 9,439 sites filtered by using FDR-adjusted binomial p-value < 0.05 as a cutoff, we observed that up to 4,592 (49%) were non-A-to-I changes (Table S9), which was significantly higher than the expectation of 7% (binomial test p < 10^-15^) based on the ratio of non-A-to-I sites to total editing sites before binomial test filtering. Similar results were also observed during our analysis of the ant datasets (Table S10). However, we did not see any improvement for the non-*Alu* sites in the human dataset, as other rigorous criteria set for non-*Alu* sites, such as the requirements for at least three different RNA reads supporting editing and an editing level greater than 10%, have overridden the effect of binomial tests (i.e. all sites passing other specific non-*Alu* site criteria have already achieved an adjusted p-value < 0.05). In conclusion, the preferential removal of non-A-to-I candidates demonstrates that binomial tests do help to reduce false positives during genome-wide identification of RNA-editing sites. However, we also acknowledge that users can raise other parameters (e.g. number of reads supporting editing, the minimal editing level) to override the effect of binomial tests at the expense of losing true editing sites with relatively weak editing signals (e.g. editing level < 10%).

**9. Analysis of human data with GIREMI**

The latest version of GIREMI (v0.2.1) was downloaded [21]. The single-nucleotide variant (SNV) list required for GIREMI was extracted from the GM12878 RNA BAM file using SAMtools' 'mpileup' function. For each SNV site, read coverage ≥ 5X was required and the variant allele (i.e. non-reference allele) was required to be supported by at least three RNA reads. Sequencing qualities on the SNV sites were required to be above 20. SNVs present in dbSNP142 [19] were marked out in the SNV list according to the GIREMI manual. Furthermore, SNV sites located in simple repeat regions, in homopolymer runs of ≥ 5 bp, or within 4 bp of a known spliced junction were removed. This refined SNV list (of 272,191 SNVs) and the RNA BAM file were then used together as inputs for GIREMI. We ran GIREMI to perform RNA-editing site identification with the following command:

giremi -f hg19.fa -l SNV.lst -o SNV.lst.out -p 1 -s 2 -m 5 RNA.bam

Instead of running each chromosome separately, GIREMI's author recommended that all chromosomes be included in a single run, to get a more accurate calculation of the mutual information distribution. The run time for GIREMI included the cumulative CPU times of generating the SNV list from the RNA-seq data using SAMtools (~9.60 h) and running GIREMI (~1.27 h). The CPU time for each step was recorded using 'runit' [22].

**10. Analysis of leaf-cutting ant and human data with REDItools**

The latest version of REDItools (v1.0.4) was downloaded [23] and installed according to the user manual. For the ant data, pre-aligned DNA and RNA reads of the nine ant samples in BAM format, generated by RES-Scanner with BWA (see Text 5 above for detail), were used as inputs. The command lines for calling editing sites (using sample L363 as an example) were as follows:

python REDItoolBlatCorrection.py -i L363_RNA.bam -f Aech.fa -F Aech.2bit -o BlatCorrection/ -V -T -Q 33 -t 10 -q 30

python REDItoolDnaRna.py -i L363_RNA.bam -j L363_DNA.bam -f Aech.fa -o ./REDItools/ -t 10 -F complete -c 10,3 -m 20,20 -q 30,30 -s2 -g1 -u -U -a 6-6 -v 3 -N 0.0 -n 0.05 -Q 33,33 -l -z -R -V -e -b BlatCorrection/ -r 6 -w splicesites_Aech.txt

We then set the required DNA variation frequencies to be 0, ≤ 0.02 and ≤ 0.05, respectively, according to the DNA information summarized for each candidate editing site by REDItools, in order to obtain three versions of final editing sites.

For the human data, pre-aligned DNA reads in BAM format downloaded from NCBI and pre-aligned RNA reads in BAM format generated by Ramaswami *et al.* [[12](#_ENREF_12)] (see Text 6 above for detail) were used as inputs. The command lines for calling *Alu* and non-*Alu* editing sites (using 'chr1' as an example) were as follows:

*Alu*:

python REDItoolDnaRna.py -i RNA_chr1.bam -j DNA_chr1.bam -f hg19.fa -o ./Alu_chr1/ -t 10 -F complete -c 10,2 -m 20,20 -s2 -g1 -u -a 6-0 -v 2 -N 0.0 -n 0.0 -V -R -e

non-*Alu*:

python REDItoolBlatCorrection.py -i RNA_chr1.bam -f hg19.fa -F hg19.2bit -o BlatCorrection_chr1/ -V -T -Q 33 -t 10

python REDItoolDnaRna.py -i RNA_chr1.bam -j DNA_chr1.bam -f hg19.fa -o ./nonAlu_chr1/ -F NOalu -Q 33,33 -c 10,2 -m 20,20 -s2 -g1 -u -a 6-0 -v 3 -N 0.0 -n 0.1 -O 0,5 -l -r 4 -w splicesites_hg19.txt -b BlatCorrection_chr1/ -V -R -e

The CPU time for each step was again recorded using 'runit'.

**Supplementary Figures**

**Figure S1.** **Correlations between editing levels estimated by TA-clonal sequencing and RNA-seq for the 76 editing sites distributed on the PCR amplicons generated by Li *et al.* [**[**9**](#_ENREF_9)**].** For each site, approximately 50 clones were randomly picked for Sanger sequencing. Fitted line indicates regressions predicting RNA-seq estimates from TA-cloning estimates.

**
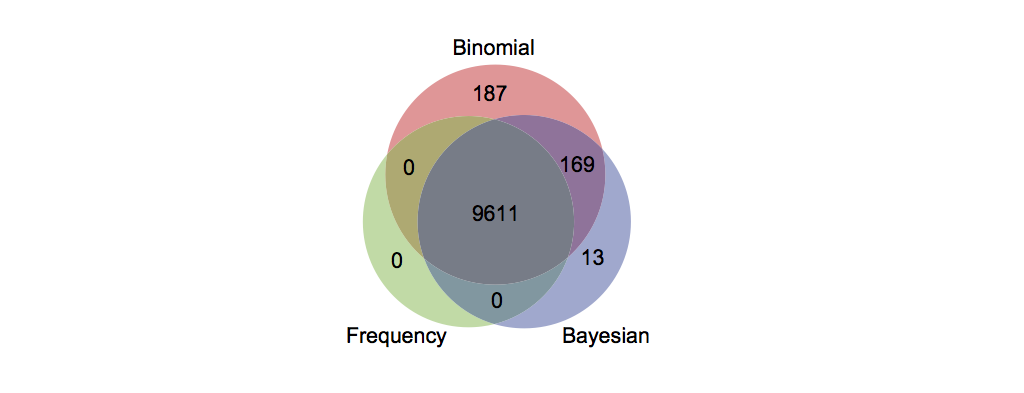
**

**Figure S2.** **Venn diagram of RNA-editing sites identified under the *Bayesian*, *Binomial* and *Frequency* homozygous genotype calling models in the sample of large workers of colony 363 (L363).** DNA- and RNA-seq data are from Li *et al.* [[9](#_ENREF_9)].


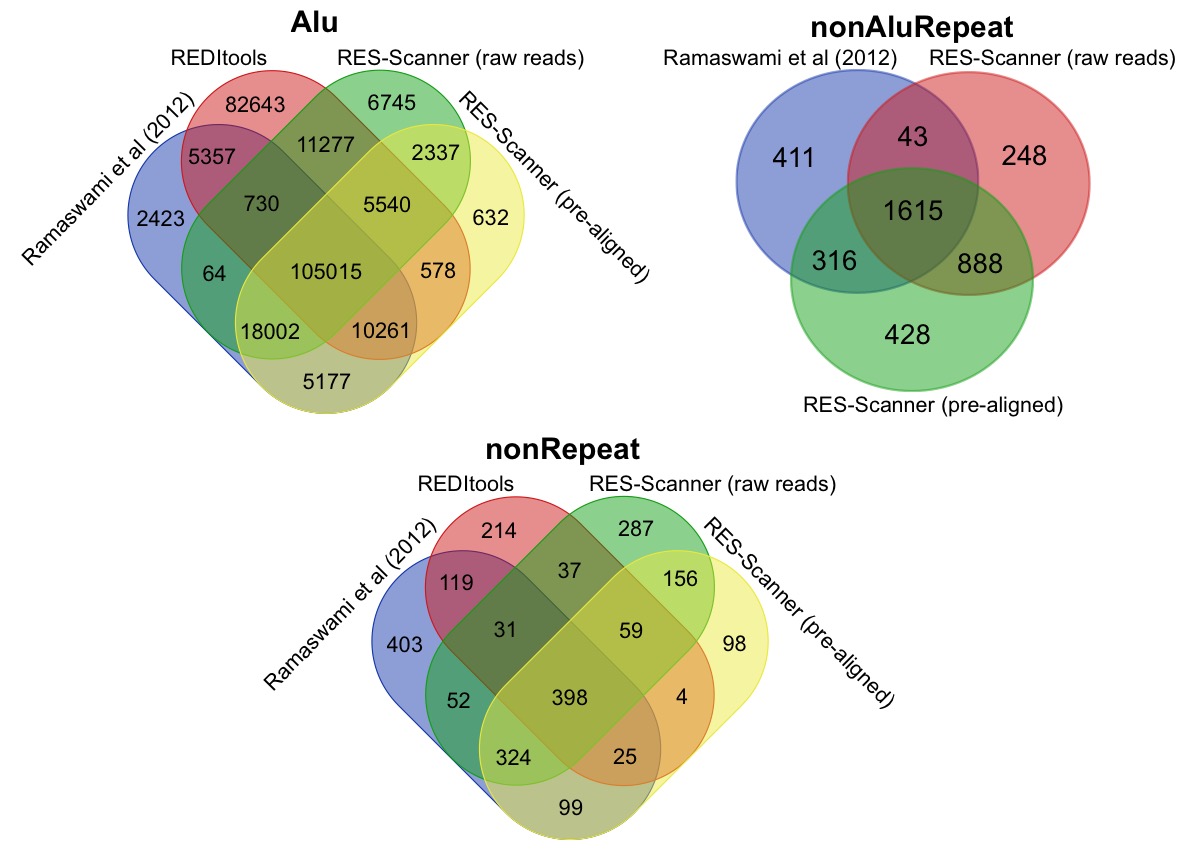


**Figure S3**. **Comparison of RNA-editing sites identified in the human GM12878 cell line from Ramaswami *et al*. [**[**12**](#_ENREF_12)**], REDItools [**[**13**](#_ENREF_13)**], RES-Scanner using pre-aligned RNA reads as inputs (pre-aligned version) and RES-Scanner using raw RNA reads as inputs (raw reads version).** Note: Picardi *et al.* [[13](#_ENREF_13)] did not provide editing sites in non-*Alu* repeat regions in their original paper on REDItools. Pre-aligned RNA reads used as inputs for RES-Scanner were originally generated in Ramaswami *et al*. [[12](#_ENREF_12)].

**Figure S4**. **Comparisons of (A) RNA read depth, (B) DNA read depth and (C) editing level between the overlapped and method-specific sites.** GM12878 RNA-editing sites from Ramaswami *et al*.[[12](#_ENREF_12)], REDItools [[13](#_ENREF_13)] and RES-Scanner (raw reads version) were first divided into two parts: the overlapping sites that are shared by at least one of the other two methods and the method-specific sites. Then read depths and editing levels were plotted for each part from each method. Sites in *Alu* repeats, non-*Alu* repeats and nonrepetitive regions were combined in this analysis. Note that Ramaswami *et al*. [[12](#_ENREF_12)] do not provide DNA depth information for their editing sites (see their Supplementary Data 1). Thus, **(B)** lacks any data from Ramaswami *et al.*

**Supplementary Tables**

**Table S1.** **Numbers and percentages of RNA-editing sites of the nine leaf-cutting ant samples identified by RES-Scanner**

| Sample* | All | A->C | A->I | A->U | C->A | C->G | C->U | G->A | G->C | G->U | U->A | U->C | U->G |
| --- | --- | --- | --- | --- | --- | --- | --- | --- | --- | --- | --- | --- | --- |
|  | **Number of editing sites** | | | | | | | | | | | | |
| G322 | 13,051 | 12 | 12,310 | 23 | 37 | 2 | 115 | 347 | 7 | 17 | 31 | 143 | 7 |
| L322 | 17,242 | 9 | 16,313 | 21 | 58 | 2 | 143 | 445 | 6 | 14 | 37 | 184 | 10 |
| S322 | 18,576 | 14 | 17,595 | 22 | 49 | 4 | 151 | 431 | 7 | 16 | 38 | 238 | 11 |
|  |  |  |  |  |  |  |  |  |  |  |  |  |  |
| G356 | 11,402 | 7 | 10,813 | 19 | 31 | 3 | 88 | 289 | 4 | 11 | 21 | 112 | 4 |
| L356 | 10,282 | 11 | 9,547 | 23 | 38 | 2 | 123 | 311 | 4 | 13 | 22 | 184 | 4 |
| S356 | 20,234 | 18 | 19,272 | 23 | 48 | 4 | 127 | 439 | 4 | 16 | 34 | 238 | 11 |
|  |  |  |  |  |  |  |  |  |  |  |  |  |  |
| G363 | 12,758 | 11 | 12,203 | 22 | 30 | 1 | 89 | 281 | 6 | 8 | 12 | 91 | 4 |
| L363 | 12,451 | 15 | 11,639 | 16 | 38 | 3 | 143 | 355 | 7 | 9 | 25 | 197 | 4 |
| S363 | 15,860 | 11 | 15,053 | 19 | 38 | 4 | 128 | 343 | 4 | 11 | 28 | 217 | 4 |
|  |  |  |  |  |  |  |  |  |  |  |  |  |  |
| Mean | 14,650 | 12 | 13,860 | 20 | 40 | 2 | 123 | 360 | 5 | 12 | 27 | 178 | 6 |
|  | **Percentage of editing sites** | | | | | | | | | | | | |
| G322 | 100 | 0.09 | 94.32 | 0.18 | 0.28 | 0.02 | 0.88 | 2.66 | 0.05 | 0.13 | 0.24 | 1.10 | 0.05 |
| L322 | 100 | 0.05 | 94.61 | 0.12 | 0.34 | 0.01 | 0.83 | 2.58 | 0.03 | 0.08 | 0.21 | 1.07 | 0.06 |
| S322 | 100 | 0.08 | 94.72 | 0.12 | 0.26 | 0.02 | 0.81 | 2.32 | 0.04 | 0.09 | 0.2 | 1.28 | 0.06 |
|  |  |  |  |  |  |  |  |  |  |  |  |  |  |
| G356 | 100 | 0.06 | 94.83 | 0.17 | 0.27 | 0.03 | 0.77 | 2.53 | 0.04 | 0.10 | 0.18 | 0.98 | 0.04 |
| L356 | 100 | 0.11 | 92.85 | 0.22 | 0.37 | 0.02 | 1.20 | 3.02 | 0.04 | 0.13 | 0.21 | 1.79 | 0.04 |
| S356 | 100 | 0.09 | 95.25 | 0.11 | 0.24 | 0.02 | 0.63 | 2.17 | 0.02 | 0.08 | 0.17 | 1.18 | 0.05 |
|  |  |  |  |  |  |  |  |  |  |  |  |  |  |
| G363 | 100 | 0.09 | 95.65 | 0.17 | 0.24 | 0.01 | 0.70 | 2.20 | 0.05 | 0.06 | 0.09 | 0.71 | 0.03 |
| L363 | 100 | 0.12 | 93.48 | 0.13 | 0.31 | 0.02 | 1.15 | 2.85 | 0.06 | 0.07 | 0.20 | 1.58 | 0.03 |
| S363 | 100 | 0.07 | 94.91 | 0.12 | 0.24 | 0.03 | 0.81 | 2.16 | 0.03 | 0.07 | 0.18 | 1.37 | 0.03 |
|  |  |  |  |  |  |  |  |  |  |  |  |  |  |
| Mean | 100 | 0.08 | 94.61 | 0.14 | 0.28 | 0.02 | 0.84 | 2.46 | 0.04 | 0.09 | 0.19 | 1.22 | 0.04 |

*G, L and S represent gynes, large workers and small workers, respectively, while 322, 356 and 363 represent colony identifiers [9].

**Table S2.** **Statistics for the pre-aligned DNA-seq reads in BAM format of the human GM12878 cell line (downloaded from the 1000 Genomes Project)**

| **Chromosome** | **Read length (bp)** | **Total reads (M)** | **Total bases (Gb)** | **Chr length (Gb)** | **Depth (X)** |
| --- | --- | --- | --- | --- | --- |
| chr1 | 36,37,44,47,51,76 | 261.81 | 12.21 | 0.25 | 49.00 |
| chr2 | 36,37,44,47,51,76 | 270.60 | 12.54 | 0.24 | 51.57 |
| chr3 | 36,37,44,47,51,76 | 212.43 | 9.83 | 0.20 | 49.65 |
| chr4 | 36,37,44,47,51,76 | 207.63 | 9.56 | 0.19 | 50.04 |
| chr5 | 36,37,44,47,51,76 | 194.05 | 8.98 | 0.18 | 49.61 |
| chr6 | 36,37,44,47,51,76 | 184.50 | 8.54 | 0.17 | 49.90 |
| chr7 | 36,37,44,47,51,76 | 174.79 | 8.10 | 0.16 | 50.91 |
| chr8 | 36,37,44,47,51,76 | 159.77 | 7.40 | 0.15 | 50.55 |
| chr9 | 36,37,44,47,51,76 | 136.11 | 6.33 | 0.14 | 44.86 |
| chr10 | 36,37,44,47,51,76 | 169.34 | 7.90 | 0.14 | 58.29 |
| chr11 | 36,37,44,47,51,76 | 147.16 | 6.85 | 0.14 | 50.71 |
| chr12 | 36,37,44,47,51,76 | 143.74 | 6.68 | 0.13 | 49.88 |
| chr13 | 36,37,44,47,51,76 | 100.29 | 4.63 | 0.12 | 40.17 |
| chr14 | 36,37,44,47,51,76 | 96.38 | 4.48 | 0.11 | 41.70 |
| chr15 | 36,37,44,47,51,76 | 90.05 | 4.20 | 0.10 | 40.97 |
| chr16 | 36,37,44,47,51,76 | 103.63 | 4.89 | 0.09 | 54.17 |
| chr17 | 36,37,44,47,51,76 | 88.38 | 4.16 | 0.08 | 51.29 |
| chr18 | 36,37,44,47,51,76 | 82.28 | 3.81 | 0.08 | 48.80 |
| chr19 | 36,37,44,47,51,76 | 65.73 | 3.11 | 0.06 | 52.55 |
| chr20 | 36,37,44,47,51,76 | 67.26 | 3.16 | 0.06 | 50.10 |
| chr21 | 36,37,44,47,51,76 | 41.16 | 1.91 | 0.05 | 39.65 |
| chr22 | 36,37,44,47,51,76 | 38.63 | 1.84 | 0.05 | 35.82 |
| chrX | 36,37,44,47,51,76 | 164.61 | 7.61 | 0.16 | 49.03 |
| chrY | 36,37,44,47,51,76 | 8.95 | 0.40 | 0.06 | 6.67 |
| chrMT | 36,37,44,47,51,76 | 8.74 | 0.44 | 0.00 | 26,273.96 |
| Whole genome | 36,37,44,47,51,76 | 3,218.02 | 149.56 | 3.09 | 48.40 |

**Table S3.** **Statistics of the pre-aligned RNA-seq reads generated in Ramaswami *et al.* [**[**12**](#_ENREF_12)**] and the raw RNA-seq reads of the human GM12878 cell line**

|  | **Forward reads (M)** | **Reverse reads (M)** | **Total reads (M)** | **Total bases (Gb)** | **Depth (X)** |
| --- | --- | --- | --- | --- | --- |
| **Pre-aligned RNA reads of GM12878** | | | | | |
| Replicate1 | 28.67 | 28.84 | 57.51 | 4.37 | 0.71 |
| Replicate2 | 31.97 | 32.86 | 64.83 | 4.93 | 0.80 |
| Merged | 60.64 | 61.70 | 122.34 | 9.30 | 1.50 |
| **Raw RNA reads of GM12878** | | | | | |
| Replicate1 | 117.88 | 117.88 | 235.76 | 7.34 | 1.19 |
| Replicate2 | 131.83 | 131.83 | 263.66 | 8.76 | 1.41 |
| Merged | 249.71 | 249.71 | 499.42 | 16.10 | 2.60 |

The lengths of both the forward and reverse reads in both replicates were 76 bp.

**Table S4. Comparison of the number of useful reads obtained by different mapping strategies focusing on the human GM12878 RNA-seq data**

| **Strategy** | **Raw reads (M)** | **Mapped reads (M)** | **Mapped reads after removing PCR duplicates (M)** |
| --- | --- | --- | --- |
| Ramaswami *et al.* [[12](#_ENREF_12)]  (BWA 'samse'; hg19 + junctions) | 499.4 | 457 | 122.3 |
| REDItools [[13](#_ENREF_13)]  (GSnap; hg19) |  | 405 | 220 |
| RES-Scanner  (BWA 'sampe'; hg19) |  | 425 | 215 |
| RES-Scanner (default)  (BWA 'sampe'; hg19 + junctions) |  | 452 | 273 |
| RES-Scanner  (HISAT2; hg19) |  | 448 | 266 |

The table shows that the performance of the default read-mapping strategy implemented in RES-Scanner is comparable to other tools, and the addition to the reference genome of exonic sequences surrounding all known splicing junctions greatly improves the number of useful reads. For those rows not relating to RES-Scanner, statistical data has been retrieved from the appropriate original papers, as indicated in the table. For GSnap used in REDItools, the figures (405 M and 220 M) represent the number of properly paired reads rather than all mapped reads [[13](#_ENREF_13)].

**Table S5**. **Numbers and percentages of RNA-editing sites in the human GM12878 cell line identified by RES-Scanner using pre-aligned RNA reads originally generated in Ramaswami *et al*. [12] as inputs**

| **Region** | **Total** | **A->C** | **A->I** | **A->U** | **C->A** | **C->G** | **C->U** | **G->A** | **G->C** | **G->U** | **U->A** | **U->C** | **U->G** |
| --- | --- | --- | --- | --- | --- | --- | --- | --- | --- | --- | --- | --- | --- |
|  | **Number of editing sites** | | | | | | | | | | | | |
| *Alu* | 147,542 | 160 | 142,170 | 312 | 649 | 132 | 718 | 584 | 165 | 1,565 | 298 | 492 | 297 |
| non*Alu*Repeat | 3,247 | 5 | 3,151 | 8 | 5 | 2 | 10 | 24 | 6 | 22 | 1 | 4 | 9 |
| nonRepeat | 1,163 | 9 | 1,018 | 3 | 7 | 2 | 12 | 64 | 7 | 19 | 2 | 9 | 11 |
| All | 151,952 | 174 | 146,339 | 323 | 661 | 136 | 740 | 672 | 178 | 1,606 | 301 | 505 | 317 |
|  | **Percentage of editing sites** | | | | | | | | | | | | |
| *Alu* | 100.00 | 0.11 | 96.36 | 0.21 | 0.44 | 0.09 | 0.49 | 0.40 | 0.11 | 1.06 | 0.20 | 0.33 | 0.20 |
| non*Alu*Repeat | 100.00 | 0.15 | 97.04 | 0.25 | 0.15 | 0.06 | 0.31 | 0.74 | 0.18 | 0.68 | 0.03 | 0.12 | 0.28 |
| nonRepeat | 100.00 | 0.77 | 87.53 | 0.26 | 0.60 | 0.17 | 1.03 | 5.50 | 0.60 | 1.63 | 0.17 | 0.77 | 0.95 |
| All | 100.00 | 0.11 | 96.31 | 0.21 | 0.44 | 0.09 | 0.49 | 0.44 | 0.12 | 1.06 | 0.20 | 0.33 | 0.21 |

**Table S6.** **Numbers and percentages of RNA-editing sites in the human GM12878 cell line identified by RES-Scanner using raw RNA reads as inputs**

| **Region** | **Total** | **A->C** | **A->I** | **A->U** | **C->A** | **C->G** | **C->U** | **G->A** | **G->C** | **G->U** | **U->A** | **U->C** | **U->G** |
| --- | --- | --- | --- | --- | --- | --- | --- | --- | --- | --- | --- | --- | --- |
|  | **Number of editing sites** | | | | | | | | | | | | |
| *Alu* | 149,710 | 193 | 143,528 | 404 | 715 | 132 | 690 | 598 | 242 | 1,786 | 456 | 576 | 390 |
| non*Alu*Repeat | 2,794 | 5 | 2,731 | 6 | 6 | 2 | 5 | 11 | 3 | 16 | 2 | 2 | 5 |
| nonRepeat | 1,344 | 15 | 1,093 | 9 | 14 | 10 | 34 | 75 | 13 | 23 | 5 | 34 | 19 |
| All | 153,848 | 213 | 147,352 | 419 | 735 | 144 | 729 | 684 | 258 | 1,825 | 463 | 612 | 414 |
|  | **Percentage of editing sites** | | | | | | | | | | | | |
| *Alu* | 100.00 | 0.13 | 95.87 | 0.27 | 0.48 | 0.09 | 0.46 | 0.40 | 0.16 | 1.19 | 0.30 | 0.38 | 0.26 |
| non*Alu*Repeat | 100.00 | 0.18 | 97.75 | 0.21 | 0.21 | 0.07 | 0.18 | 0.39 | 0.11 | 0.57 | 0.07 | 0.07 | 0.18 |
| nonRepeat | 100.00 | 1.12 | 81.32 | 0.67 | 1.04 | 0.74 | 2.53 | 5.58 | 0.97 | 1.71 | 0.37 | 2.53 | 1.41 |
| All | 100.00 | 0.14 | 95.78 | 0.27 | 0.48 | 0.09 | 0.47 | 0.44 | 0.17 | 1.19 | 0.30 | 0.40 | 0.27 |

**Table S7. Statistics for non-synonymous and synonymous editing sites in different studies**

| **Dataset** | **Region** | **A->I** | | | | **non A->I** | | | |
| --- | --- | --- | --- | --- | --- | --- | --- | --- | --- |
|  |  | **Nonsyn.** | **Syn.** | **Total** | **Nonsyn. ratio** | **Nonsyn.** | **Syn.** | **Total** | **Nonsyn. ratio** |
| Ramaswami *et al.* [[12](#_ENREF_12)] | *Alu* | 234 | 128 | 362 | 0.65 | 19 | 6 | 25 | 0.76 |
|  | non*Alu*Repeat | 3 | 2 | 5 | 0.60 | 0 | 0 | 0 | 0.00 |
|  | nonRepeat | 4 | 0 | 4 | 1.00 | 8 | 4 | 12 | 0.67 |
|  | All | 241 | 130 | 371 | 0.65 | 27 | 10 | 37 | 0.73 |
| REDItools [[13](#_ENREF_13)] | *Alu* | 367 | 193 | 560 | 0.66 | 52 | 17 | 69 | 0.75 |
|  | non*Alu*Repeat | Not investigated | | | | | | | |
|  | nonRepeat | 2 | 0 | 2 | 1.00 | 5 | 4 | 9 | 0.56 |
|  | All | 369 | 193 | 562 | 0.66 | 57 | 21 | 78 | 0.73 |
| RES-Scanner  (pre-aligned) | *Alu* | 229 | 124 | 353 | 0.65 | 14 | 4 | 18 | 0.78 |
|  | non*Alu*Repeat | 5 | 2 | 7 | 0.71 | 2 | 0 | 2 | 1.00 |
|  | nonRepeat | 1 | 0 | 1 | 1.00 | 9 | 8 | 17 | 0.53 |
|  | All | 235 | 126 | 361 | 0.65 | 25 | 12 | 37 | 0.68 |
| RES-Scanner  (raw reads) | *Alu* | 222 | 120 | 342 | 0.65 | 35 | 7 | 42 | 0.83 |
|  | non*Alu*Repeat | 3 | 2 | 5 | 0.60 | 3 | 0 | 3 | 1.00 |
|  | nonRepeat | 3 | 1 | 4 | 0.75 | 15 | 11 | 26 | 0.58 |
|  | All | 228 | 123 | 351 | 0.65 | 53 | 18 | 71 | 0.75 |

**Table S8. Comparison of RNA-editing sites detected by RES-Scanner and REDItools**

| **Sample** | **RES-Scanner** | | **REDItools** | | | | | |
| --- | --- | --- | --- | --- | --- | --- | --- | --- |
|  |  |  | **DNA_VF = 0** | | **DNA_VF** **≤ 0.02** | | **DNA_VF ≤ 0.05** | |
|  | **Total** | **% A>I** | **Total** | **% A>I** | **Total** | **% A>I** | **Total** | **% A>I** |
| G322 | 13,051 | 94.32 | 10,950 | 75.48 | 10,984 | 75.28 | 11,646 | 72.02 |
| L322 | 17,242 | 94.61 | 15,917 | 86.42 | 15,972 | 86.21 | 17,057 | 81.91 |
| S322 | 18,576 | 94.72 | 20,397 | 83.39 | 20,445 | 83.24 | 21,365 | 80.52 |
|  |  |  |  |  |  |  |  |  |
| G356 | 11,402 | 94.83 | 9,221 | 75.91 | 9,244 | 75.76 | 9,757 | 73.00 |
| L356 | 10,282 | 92.85 | 8,685 | 71.36 | 8,734 | 71.10 | 9,281 | 68.25 |
| S356 | 20,234 | 95.25 | 21,968 | 88.92 | 22,114 | 88.62 | 22,873 | 86.74 |
|  |  |  |  |  |  |  |  |  |
| G363 | 12,758 | 95.65 | 9,846 | 82.21 | 9,878 | 82.02 | 10,255 | 80.08 |
| L363 | 12,451 | 93.48 | 10,113 | 78.91 | 10,139 | 78.82 | 10,537 | 76.94 |
| S363 | 15,860 | 94.91 | 16,709 | 81.62 | 16,739 | 81.51 | 17,193 | 80.13 |
|  |  |  |  |  |  |  |  |  |
| Mean | 14,650 | 94.61 | 13,756 | 80.47 | 13,805 | 80.28 | 14,440 | 77.73 |

Some basic parameters, including DNA depth ≥ 10, RNA depth ≥ 3 and editing level ≥ 5% were set the same for both software packages (see command lines below for more detail). For RES-Scanner, we specifically required the Bayesian posterior probability for defining homozygous genotypes to be > 0.95 and the FDR-adjusted binomial test p-value for defining editing sites to be < 0.05. For REDItools, we arbitrarily set the maximum frequency of non-reference bases present in the DNA-seq data (DNA variation frequency, or DNA_VF) to be 0, ≤ 0.02 and ≤ 0.05, and thus generated three versions of results.

The RES-Scanner command for detecting editing sites on the nine leaf-cutting ant samples:

- perl RES-Scanner_identification.pl --config RES_Scanner_identification_config.txt --out ./RES_Scanner_identification_outdir/ --genome Aech.fa -ss 1 --samtools /usr/bin/samtools-0.1.18/samtools --blat /usr/bin/blat_64/blat --trim 6,6 --q 30 --mq 20 --ploidy 8 --rmdup 1 --paralogous_R 1 --paralogous_D 1 --homopolymer 1 --posdir /usr/bin/Aech/posdir/

The REDItools (v1.0.4) commands for detecting editing sites on the nine leaf-cutting ant samples:

- python REDItoolBlatCorrection.py -i RNA.bam -f Aech.fa -F Aech.2bit -o BlatCorrection/ -V -T -Q 33 -t 10 -q 30
- python REDItoolDnaRna.py -i RNA.bam -j DNA.bam -f Aech.fa -o ./REDItools/ -t 10 -F complete -c 10,3 -m 20,20 -q 30,30 -s2 -g1 -u -U -a 6-6 -v 3 -N 0.0 -n 0.05 -Q 33,33 -l -z -R -V -e -b BlatCorrection/ -r 6 -w splicesites_Aech.txt

We then required the DNA variation frequencies to be 0, ≤ 0.02 and ≤ 0.05, respectively, according to the DNA information summarized for each candidate editing site by REDItools, to obtain three versions of the final editing sites.

**Table S9. Percentage of non-A-to-I editing sites detected by RES-Scanner in the human GM12878 dataset (raw reads version) before and after** **using binomial test filtering to distinguish RNA-editing sites from sequencing errors**

| **Region** | **Before filtering** | | **After filtering** | | **Filtered sites** | | **P-value** |
| --- | --- | --- | --- | --- | --- | --- | --- |
|  | **Total** | **% non-A-to-I** | **Total** | **% non-A-to-I** | **Total** | **% non-A-to-I** |  |
| *Alu* | 159,149 | 6.77 | 149,710 | 4.13 | 9,439 | 48.65 | <10^-15^ |
| non*Alu*Repeat | 2,794 | 2.25 | 2,794 | 2.25 | 0 | 0.00 | N/A |
| nonRepeat | 1,344 | 18.68 | 1,344 | 18.68 | 0 | 0.00 | N/A |

An FDR-adjusted binomial p-value < 0.05 was chosen as the cutoff for the filtering. The p-value in the last column was calculated according to a two-sided binomial test with '% non-A-to-I' before filtering (e.g. 6.77% for *Alu*) as the hypothesized probability of success, the observed number of non-A-to-I sites among the filtered sites (e.g. 48.65% of 9,439 = 4,592 for *Alu*) as the number of successes, and the total number of filtered sites (e.g. 9,439 for *Alu*) as the number of trials, showing that for *Alu* the observed non-A-to-I percentage for the filtered sites (48.65%) was significantly higher than expected (6.77%).

**Table S10. Percentage of non-A-to-I editing sites detected by RES-Scanner in the leaf-cutting ant dataset before and after using binomial test filtering to distinguish RNA-editing sites from sequencing error**

| **Sample** | **Before filtering** | | **After filtering** | | **Filtered sites** | | **P-value** |
| --- | --- | --- | --- | --- | --- | --- | --- |
|  | **Total** | **% non-A-to-I** | **Total** | **% non-A-to-I** | **Total** | **% non-A-to-I** |  |
| G322 | 13,514 | 6.25 | 13,051 | 5.68 | 463 | 22.25 | <10^-15^ |
| L322 | 17,643 | 5.88 | 17,242 | 5.39 | 401 | 27.18 | <10^-15^ |
| S322 | 18,943 | 5.75 | 18,576 | 5.28 | 367 | 29.43 | <10^-15^ |
|  |  |  |  |  |  |  |  |
| G356 | 11,964 | 6.02 | 11,402 | 5.17 | 562 | 23.31 | <10^-15^ |
| L356 | 10,697 | 7.74 | 10,282 | 7.15 | 415 | 22.41 | <10^-15^ |
| S356 | 20,627 | 5.30 | 20,234 | 4.75 | 393 | 33.33 | <10^-15^ |
|  |  |  |  |  |  |  |  |
| G363 | 13,327 | 4.97 | 12,758 | 4.35 | 569 | 18.98 | <10^-15^ |
| L363 | 12,865 | 7.10 | 12,451 | 6.52 | 414 | 24.64 | <10^-15^ |
| S363 | 16,240 | 5.69 | 15,860 | 5.09 | 380 | 30.79 | <10^-15^ |

FDR-adjusted binomial p-value < 0.05 was chosen as the cutoff for the filtering. See explanation in Table S9 for more details.

**Supplementary References**

1. Hodgkinson A, Eyre-Walker A. Human triallelic sites: evidence for a new mutational mechanism? Genetics. 2010;184:233-41.

2. Keller I, Bensasson D, Nichols RA. Transition-transversion bias is not universal: a counter example from grasshopper pseudogenes. PLoS Genet. 2007;3:e22.

3. Balding DJ, Bishop MJ, Cannings C. Handbook of statistical genetics, 3rd edn. Chichester, UK: John Wiley & Sons; 2007.

4. Rosner B. Fundamentals of biostatistics, 7th edn. Boston, MA: Cengage Learning; 2010.

5. Dohm JC, Lottaz C, Borodina T, Himmelbauer H. Substantial biases in ultra-short read data sets from high-throughput DNA sequencing. Nucleic Acids Res. 2008;36:e105.

6. Kircher M, Stenzel U, Kelso J. Improved base calling for the Illumina Genome Analyzer using machine learning strategies. Genome Biol. 2009;10:R83.

7. Li R, Li Y, Fang X, Yang H, Wang J, Kristiansen K, et al. SNP detection for massively parallel whole-genome resequencing. Genome Res. 2009;19:1124-32.

8. Benjamini Y, Drai D, Elmer G, Kafkafi N, Golani I. Controlling the false discovery rate in behavior genetics research. Behav Brain Res. 2001;125:279-84.

9. Li Q, Wang Z, Lian J, Schiøtt M, Jin L, Zhang P, et al. Caste-specific RNA editomes in the leaf-cutting ant Acromyrmex echinatior. Nat Commun. 2014;5:4943.

10. Hughes WO, Boomsma JJ. Genetic royal cheats in leaf-cutting ant societies. Proc Natl Acad Sci U S A. 2008;105:5150-53.

11. Djebali S, Davis CA, Merkel A, Dobin A, Lassmann T, Mortazavi A, et al. Landscape of transcription in human cells. Nature. 2012;489:101-8.

12. Ramaswami G, Lin W, Piskol R, Tan MH, Davis C, Li JB. Accurate identification of human Alu and non-Alu RNA editing sites. Nat Methods. 2012;9:579-81.

13. Picardi E, Pesole G. REDItools: high-throughput RNA editing detection made easy. Bioinformatics. 2013;29:1813-14.

14. NCBI Gene Expression Omnibus [http://www.ncbi.nlm.nih.gov/geo/]. Accessed 24 Jul 2016.

15. Genomic Resources for Acromyrmex echinatior [http://hymenopteragenome.org/acromyrmex/]. Accessed 24 Jul 2016.

16. 1000 Genomes Project GM12878 Cell Line Database [ftp://ftp-trace.ncbi.nih.gov/1000genomes/ftp/technical/pilot2_high_cov_GRCh37_bams/data/NA12878/alignment/]. Accessed 24 Jul 2016.

17. Yates A, Akanni W, Amode MR, Barrell D, Billis K, Carvalho-Silva D, et al. Ensembl 2016. Nucleic Acids Res. 2016;44;D710-16.

18. Genome Reference Consortium Human Reference 37 (hg19) Assembly (Feb 2009) [http://hgdownload.cse.ucsc.edu/goldenPath/hg19/bigZips/ and http://hgdownload.cse.ucsc.edu/goldenPath/hg19/database/]. Accessed 24 Jul 2016.

19. UCSC Genome Bioinformatics Human Genome Build 38 (Dec 2013) [http://hgdownload.cse.ucsc.edu/goldenPath/hg38/database/snp142.txt.gz]. Accessed 24 Jul 2016.

20. UCSC Long RNA-seq from ENCODE/Cold Spring Harbor Lab [http://hgdownload.cse.ucsc.edu/goldenpath/hg19/encodeDCC/wgEncodeCshlLongRnaSeq/]. Accessed 24 Jul 2016.

21. GIREMI [https://github.com/zhqingit/giremi]. Accessed 24 Jul 2016.

22. Runit - a Unix init scheme with service supervision [http://smarden.org/runit/]. Accessed 24 Jul 2016.

23. REDItools: RNA editing detection by NGS data [https://sourceforge.net/projects/reditools/]. Accessed 24 Jul 2016.
