# Supplementary material for: RES-Scanner: a software package for genome-wide identification of RNA-editing sites
Source: Gigascience. 2016 Aug 18;5:37. doi: 10.1186/s13742-016-0143-4 (PMC4989487; doi:10.1186/s13742-016-0143-4)
Supplement: Additional file 2: — User manual for RES-Scanner. (DOCX 70 kb) [file 13742_2016_143_MOESM2_ESM.docx]

**User Manual**

**Introduction**

RES-Scanner is a software package written in the Perl programming language that detects and annotates RNA-editing sites using matched RNA-seq and DNA-seq data from the same individuals or samples. RES-Scanner allows the use of both raw high-throughput sequencing (HTS) reads and pre-aligned reads in BAM format as inputs. When inputs are HTS reads, RES-Scanner will invoke the BWA mapping package to align reads to the reference genome automatically. To rigorously discriminate potential false positives resulting from genetic variants, we have equipped RES-Scanner with sophisticated statistical models to infer the reliability of homozygous genotypes called from DNA-seq data. These models are applicable to samples from either a single individual or a pool of multiple individuals if the ploidy information is known. In addition, RES-Scanner implements statistical tests to distinguish genuine RNA-editing sites from sequencing errors, and provides a series of sophisticated filters to remove false positives resulting from mapping errors. Finally, RES-Scanner can improve the completeness and accuracy of editing site identification when the data of multiple samples are available.

**Some important processes and parameters implemented in RES-Scanner**

A major challenge in RNA-editing identification using HTS data is to distinguish RNA-editing sites from genome-encoded SNPs and technical artefacts caused by sequencing or read-mapping errors. RES-Scanner conducts rigorous statistical analyses and stringent filtering as described below to reduce false positives in RNA-editing sites.

- **Mismatch number/rate for read alignment (option --n).** As RNA-editing sites tend to occur in clusters in metazoa, which will result in more mismatches between RNA reads and the reference genome, we recommend allowing for a larger mismatch number/rate during BWA alignment. The default mismatch rate used by RES-Scanner is 0.08, twice the default value of 0.04 recommended by BWA for normal read alignment. Users can modify the default setting via option --n.
- **Ambiguously mapped reads.** Three steps are taken to reduce ambiguously mapped reads: (1) reads that mapped to multiple locations or could be mapped to another location when allowing slightly more mismatches are removed by force. Only the reads mapped uniquely on one location of the reference genome with no suboptimal hits are kept for further analysis. (2) For paired-end reads, the mapped read pairs with incorrect direction will be filtered by force. (3) The default value of BWA mapping quality score (option --mq) is set to 20 by RES-Scanner, a cutoff which is widely used by other studies to reduce ambiguously mapped reads. Users can choose a larger cutoff using the option --mq.
- **PCR duplication.** Reads resulting from PCR duplication during DNA/RNA library construction will increase the coverage of genomic sites artificially, and may affect the calling of homozygous genotypes and RNA-editing sites. Thus, duplicated reads - except that with the highest mapping quality - can be removed by RES-Scanner (option --rmdup) by invoking the 'rmdup' utility of SAMtools.
- **Read trimming (option --trim).** Given the higher error rate of Illumina sequencing at the ends of reads, the introduction of mismatches at the 5' read ends by random-hexamer priming during the first- and second-strand syntheses of RNA library construction, and the mapping errors at both the 5' and 3' ends resulting from the incorrect handling of insertions/deletions, false positives in RNA-editing sites are always disproportionately increased in both ends of the reads. Thus, RES-Scanner truncates six bases from both ends of each read by default. Users can change the number of bases truncated with option --trim.
- **Strand-specific mode (option --ss).** RES-Scanner supports the RNA-seq data sequenced from the non-strand-specific or strand-specific libraries under the dUTP protocol. Users need to specify the library type for their RNA-seq data using the option --ss.
- **Homozygous genotype calling (option --method).** A major source of false positives in RNA-editing sites is undetected SNPs. RES-Scanner provides three models for calling homozygous genomic sites from the DNA-seq data: *Bayesian*, *Binomial* and *Frequency*. In practice, the *Frequency* model runs faster than the *Binomial* model, and the *Binomial* model runs much faster than the *Bayesian* model. However, only the *Bayesian* model simultaneously considers the depth and quality of RNA bases covering a genomic site. If the average depth of the DNA-seq data is low (e.g. ≤ 10X) or particularly high (e.g. ≥ 50X), we recommend selection of the *Bayesian* or *Binomial* models to statistically estimate the genomic homozygosity for the candidate editing sites. This is expected to reduce false positives when DNA-seq depth is low and reduce false negatives when DNA-seq depth is high.
- **Known SNPs.** RES-Scanner also allows users to supply a list of potential SNPs derived from other analyses or databases of the target species, and excludes candidate editing sites that overlap these SNPs using the option --knownSNP.
- **The prior probability for a genomic position to be homozygous (option -HomoPrior) and transition/transversion ratio (****option --rate).** In the *Bayesian* model for calling homozygous genomic sites, these two parameters are required for calculating the prior probability of each possible genotype for a genomic site. The prior probability of homozygous genomic positions (option -HomoPrior) ranges from zero to one, and can be easily obtained by counting the homozygous and heterozygous genomic sites in an SNP-calling analysis (e.g. GATK) using the DNA-seq data, followed by calculation of the ratio of homozygous sites over all of the called genomic sites. In general, and based on our previous surveys in a wide range of species, the value is seldom lower than 0.99 for a sample from a diploid individual. For nuclear genes, the transition/transversion ratio may be slightly different among species and even among different parts of a genome, and typical estimations of transition/transversion ratio range from 1.5 to 5, based on previous reports. In this instance, we recommend a value of 2 as the default transition/transversion ratio across the entire genome. However, users are able to specify their own transition/transversion ratio with the option --rate.
- **Sequencing depth (options --DNAdepth and --RNAdepth).** Insufficient sequencing depth may result in a high false positive rate in calling homozygous genotypes and RNA-editing sites. By default, RES-Scanner requires at least ten DNA reads for calling a homozygous genomic site and at least three RNA reads for calling an RNA-editing site. The RNA read depth is set to three as a default, as RES-Scanner also requires several more criteria for the RNA reads supporting RNA editing (see below). However, users can easily choose higher DNA/RNA depth cutoffs by using the options --DNAdepth and --RNAdepth.
- **Phred-scaled base** **quality score (option --q).** A low base quality score is associated with high rates of sequencing error. There are several commonly used base quality cutoffs, namely 20, 25 and 30, which correspond to 1%, 0.3% and 0.1% error rates of base calling, respectively. RES-Scanner applies a quality cutoff of 30 by default.
- **RNA reads supporting editing (options --editDepth and --editLevel)**. RES-Scanner requires that a candidate editing site must be supported by: (1) at least three RNA reads that are mapped to overlapping but not identical positions in the reference genome (option --editDepth); (2) having an editing level ≥ 0.05 (option --editLevel); (3) at least one RNA read in the middle of its length (e.g. between positions 23 and 68 of a 90-bp read), to avoid a candidate editing site only being supported by the near-end regions of RNA reads, which have a higher probability of being the result of mapping error.
- **Homopolymers (option --homopolymer).** RES-Scanner can remove candidate editing sites in homopolymer runs of five or more base pairs (e.g. AAAAA), as homopolymers are known to have higher rates of sequencing error. Users can activate this function with the option --homopolymer.
- **Intronic candidate sites close to splice junctions (option --intronic).** As RNA reads are generally sequenced from mature mRNA with introns removed, RNA reads covering a splice site will have a higher probability of mapping error in the intronic site. Thus, by default, RES-Scanner can remove intronic candidate editing sites occurring within six bases of a splice site. Users can disable this function by setting the value of option --intronic to 0.
- **Candidate editing sites in similar paralogous regions (options --paralogous_R and --paralogous_D).** To avoid potential false positives resulting from mis-mapping of reads between very similar paralogous regions, RES-Scanner invokes BLAT to re-align all the reads that support RNA editing (i.e. reads showing a mismatch from the reference). Then a read is defined as a qualifying read if the best hit of this read overlaps its original candidate site and the second-best hit, if it exists, has a BLAT score < 95% of the best hit. Only candidate editing sites with a proportion of qualifying reads relative to all BLAT-realigned reads exceeding a given cutoff (default 0.5, the larger the stricter) are kept. This function is activated by options --paralogous_R and --blat. In addition, RES-Scanner can further discard candidate editing sites with DNA read depths of more than twice the genome-wide peak or mean depth, as such sites are likely to be located in regions showing copy number variation (i.e. another kind of highly similar paralogous region that is not fully present in the reference genome). This function is activated via option --paralogous_D.
- **Candidate editing sites resulting from sequencing error (option --editPvalue).** To avoid false positives due to sequencing errors, RES-Scanner performs statistical tests for all candidate editing sites based on the binomial distribution. P-values are adjusted by Benjamini-Hochberg false discovery rates (FDRs) and it is recommended that an FDR below 0.05 or 0.01 be chosen as the significant threshold.

**Requirements**

1. RES-Scanner is written in the Perl language and requires pre-installation of some Perl modules and software packages:

- Perl modules: File::Basename; Cwd; Getopt::Long; FindBin.
- BWA (http://bio-bwa.sourceforge.net/)
- SAMtools (http://samtools.sourceforge.net/)
- BLAT (http://genome.cshlp.org/content/12/4/656.full)

BWA version 0.5.9-r16 and SAMtools version 0.1.18 were used for testing during our development. BWA is required by the RES-Scanner alignment pipeline and BLAT is optional for the RES-Scanner identification pipeline.

1. To annotate the identified RNA-editing sites, users need to activate the option --posdir by providing a directory containing the position files of relevant genomic features. Annotation files should be given meaningful names of the form *FeatureName*.pos (e.g. 5UTR.pos, CDS.pos, intron.pos, 3UTR.pos, ncRNA.pos, repeat.pos). If a file with the name CDS.pos is provided, the function of inferring the codon and amino acid change after RNA editing is activated by default. If a file with the name intron.pos is made available, the splicing site information can be obtained from the location of each intron, which is used by the RES-Scanner alignment pipeline to generate exonic sequences surrounding known splicing junctions. This is activated by option --junction, and the function of removing intronic candidate editing sites close to splicing junctions can be activated by option --intronic in the RES-Scanner identification pipeline.

The format of a *FeatureName*.pos file is:

- Position ID (Note: For genic features - that is, 5UTR, CDS, intron and 3UTR - if one gene has multiple transcripts, the Position ID should be given as the transcript ID or protein ID followed by an underscore, the corresponding feature symbol and the serial number of the feature. For example, if the protein ID is Aech_16644, for its first CDS the position ID is assigned as Aech_16644_CDS-N1. However, for other non-genic features with no internal structure, the only requirement for the Position ID is that it must be unique.)
- Chromosome ID
- Strand
- Start position
- End position.

Here is an example of a CDS.pos file:

Aech_16644_CDS-N1 scaffold943 + 39233 39843

Aech_16644_CDS-N2 scaffold943 + 39926 40571

Aech_16644_CDS-N3 scaffold943 + 40671 40970

Aech_16645_CDS-N1 scaffold943 - 42074 42183

Aech_16645_CDS-N2 scaffold943 - 41734 42002

Aech_16645_CDS-N3 scaffold943 - 41441 41642

And here is an example for a repeat.pos file:

TE070753-repbase scaffold943 + 6681 6742

TE070754-repbase scaffold943 + 7814 7827

TE070756-repbase scaffold943 + 7841 7948

TE070758-repbase scaffold943 - 14384 14485

TE070760-repbase scaffold943 - 14511 14539

If an external SNP list supplied by users is in GFF3 format, only the position information (columns 1, 4 and 5) is used:

chr3 snp_hg19 snp 131051 131051 . + . ID=rs530609942;Type=C/G;

chr3 snp_hg19 snp 131052 131052 . + . ID=rs374590652;Type=C/T;

**Installation**

RES-Scanner is a standalone package. To install the package, users can simply download the latest version from https://github.com/ZhangLabSZ/RES-Scanner and decompress it. The pipeline has two elements: **alignment**, which is optional, followed by **identification**. Before running the pipeline, please make sure that the requirements described above have been met. We have tested our pipeline successfully on Linux and Mac OS X Server.

**RES-Scanner alignment: options and usage (optional)**

The scripts RES-Scanner_alignment.part1.pl and RES-Scanner_alignment.part2.pl are used to align the DNA/RNA reads to the reference genomes and generate BAM files automatically. However, we recognize that the default mapping strategy implemented in RES-Scanner cannot be optimal in all cases, so this alignment step is optional. RES-Scanner also supports users by accepting pre-aligned DNA and RNA reads in BAM format from other aligners, such as Bowtie 2, TopHat2, GSnap, and HISAT2, and inputting them directly to the RES-Scanner identification pipeline.

The alignment pipeline involves two parts, the first of which is optional. These parts, together with the pipeline's inputs and outputs are described below.

**Input**

A configuration file containing the RNA-seq and DNA-seq data is the main input file and must be manually created by users. The configuration file is a tab-delimited table with five or six columns as follows:

- Tag: 'DNA' or 'RNA'
- Sample ID
- Lane ID
- The maximum insert size (bp) of paired-end reads, or the read length of single-end reads. The maximum insert size is required by BWA in paired-end mapping mode. For RNA-seq read mapping, due to the existing splicing events, the actual insert size between paired reads may vary considerably, so we recommend that a big enough value is set based on the largest intron size of the studied species (e.g. 100,000 bp for ant and 1,500,000 bp for human).
- Absolute path of file 1.fastq.
- Absolute path of file 2.fastq (optional); this column can be absent for single-end sequencing data.

Examples of configuration files for each of the data options are presented below, starting with **paired-end DNA-seq** data:

DNA Queen DNA-seq_LaneA 200 /Absolute_path/LaneA.1.fastq /Absolute_path/LaneA.2.fastq

DNA worker DNA-seq_LaneB 250 /Absolute_path/LaneB.1.fastq /Absolute_path/LaneB.2.fastq

Sample configuration file for **single-end DNA-seq** data:

DNA Queen DNA-seq_LaneA 90 /Absolute_path/LaneA.1.fastq

DNA worker DNA-seq_LaneB 45 /Absolute_path/LaneB.1.fastq

Sample configuration file for **paired-end RNA-seq** data:

RNA Queen RNA-seq_LaneA 100000 /Absolute_path/LaneA.1.fastq /Absolute_path/ LaneA.2.fastq

RNA worker RNA-seq_LaneB 90000 /Absolute_path/LaneB.1.fastq /Absolute_path/ LaneB.2.fastq

Sample configuration file for **single-end RNA-seq** data:

RNA Queen RNA-seq_LaneA 90 /Absolute_path/LaneA.1.fastq

RNA worker RNA-seq_LaneB 45 /Absolute_path/LaneB.1.fastq

**Steps - Part 1**

1. Index the reference genome for BWA alignment (optional).
2. Preprocess the FASTQ files before BWA alignment. As the memory requirement is positively correlated with the size of a FASTQ file, the FASTQ file can be split into several files of no more than 3 Gb per file to limit memory usage and speed up the alignment step (optional).

Options (default values in square brackets):

--ref *FILE* The reference genome in FASTA format.

--outDir *STR* The output directory [./].

--index *NUM* Index the reference genome for BWA: '1' for yes, '0' for no, default yes [1].

--junction *FILE* The file with junction information in POS format (i.e. intron.pos). Force --index

[null]. (Note: The length of all RNA-seq reads should stay the same within a FASTQ file pair.)

--readlen *INT* The length (bp) of RNA-seq reads for --junction option [null].

--bwa *FILE* The absolute path of the BWA package pre-installed in the local machine.

--config *FILE* The configuration file that contains the DNA-seq and RNA-seq data (see Input above).

--split Split FASTQ files for multithreading [null].

--run  Run the jobs in serial working mode (i.e. automatically run the jobs one by one).

--help Show the help information.

Usage:

perl RES-Scanner_alignment.part1.pl --outDir ./outdir/ --ref reference.fa --bwa /path_to_bwa/bwa --index 1 --config config.file --junction intron.pos --split

Note: After executing Part 1, one (--index 0) or two (--index 1) shell scripts will be generated in the directory ./outdir/. If option --run is not activated, users must run the scripts manually, in order, with the commands sh ./outdir/step0.sh and sh ./outdir/step1.sh or else submit the scripts as jobs to computing servers. It should be noted that the step1.sh script may contain multiple parallel command lines, and we recommend that users run these command lines in parallel by submitting them as jobs to computing servers simultaneously. If option --run is activated, all the jobs in Part 1 will be run one by one automatically. Before proceeding to Part 2, please make sure that all current jobs have completed successfully.

**Steps - Part 2**

1. Call BWA for read mapping.
2. Merge BAM files for each sample.

Options (default values in square brackets):

--ref *FILE* Index of reference; please input the absolute path.

--outDir *STR* The output directory; must be the same as that of Part 1 [./].

--n *NUM* Maximum mismatch number (int) or rate (float) for read alignment, required by BWA [0.08].

--k *INT* Maximum mismatch number in the seed sequence for read alignment, required by BWA [3].

--l *INT* Length of seed sequence for read alignment, required by BWA [32].

--t *INT* Number of threads for BWA mapping [1].

--config *FILE* The configuration file that contains the DNA-seq or RNA-seq data.

--bwa *FILE* The absolute path of the BWA package pre-installed in the local machine.

--samtools *FILE* The absolute path of the SAMtools package pre-installed in the local machine.

--index *NUM* Reference genome for BWA indexed in Part 1: '1' for yes, '0' for no [1].

--junction *FILE* The file with junction information in POS format (i.e. intron.pos). Force --index [null].

--run Run the jobs in serial working mode (i.e. automatically run the jobs one by one).

--help Show the help information.

Usage:

perl RES-Scanner_alignment.part2.pl --config config.file --ref reference.fa --outDir ./outdir --bwa /path_to_bwa/bwa --samtools /path_to_samtools/samtools --index 1

Note: Before initiating Part 2, please make sure that all jobs in Part 1 have finished successfully. After executing the command line of Part 2, two shell scripts, named step2.sh and step3.sh, will be generated in the directory ./outdir/. If option --run is not activated, users must manually run the scripts in order with the commands sh ./outdir/step2.sh and sh ./outdir/step3.sh or else submit the scripts as jobs to computing servers. It should be noted that each of these scripts may contain multiple parallel command lines, and we recommend that users run these command lines in parallel by submitting them as jobs to computing servers simultaneously. If option --run is activated, all the jobs in Part 2 will be run one by one automatically. Before proceeding to the identification pipeline, please make sure that all current jobs have completed successfully.

**Output**

The final alignment files in BAM format are stored in the designated directory of each sample under ./outdir/sampleID/. If a sample has multiple lanes of sequencing data, data from multiple lanes will be merged according to sample.

**RES-Scanner identification: options and usage**

**Input**

Before running the RES-Scanner identification pipeline, users should create a configuration file that provides the sample information for the DNA and RNA BAM files. If the BAM files are generated by the RES-Scanner alignment pipeline described above, this configuration file will be generated automatically. The configuration file is a tab-delimited table with three columns as follows:

- Sample ID, which must be unique for each line
- Absolute path of BAM file for DNA data alignment
- Absolute path of BAM file for RNA data alignment.

An example of the configuration file:

Queen /Absolute_path/queen_DNA.bam /Absolute_path/queen_RNA.bam

Worker /Absolute_path/worker_DNA.bam /Absolute_path/worker_RNA.bam

**Steps**

The RES-Scanner identification pipeline involves four steps: steps 1 and 2 are mainly to filter low-quality mapping data and PCR duplications in BWA alignments; step 3 is used for detecting candidate editing sites based on multiple criteria; step 4 is used for performing advanced filtering to further reduce false positives and retrieve some missed editing sites when multiple samples are available, as a number of true editing sites may have been missed due to the stringent filtering criteria applied in step 3.

Options (default values in square brackets):

--config *FILE* Tab-delimited configuration file with three columns (see details in Input above)*.*

--out *STR* The output directory.

--genome *FILE* Reference genome.

--ss *NUM* Strand-specific RNA-seq data; '1' for yes, '0' for no . Note: Only strand-specific RNA-seq library generated by the dUTP protocol is currently supported [1].

--trim *INT* The number of bases self-clipped at 5' and 3' ends of a read, respectively [6,6].

--mis *NUM* The maximum number of mismatches allowed in a read alignment [5].

--q *NUM* Phred-scaled base quality score cutoff [30].

--mq *NUM* Mapping quality score cutoff [20].

--phred *NUM* Encoding methods of Phred quality score for reads in DNA.bam and RNA.bam files, respectively. For example, [64,33] means that DNA is encoded by ASCII-64 and RNA is encoded by ASCII-33 [33,33].

--DNAdepth *INT* The minimum depth of DNA reads required by a candidate editing site. A genomic site covered by less than this depth will be filtered [10].

--RNAdepth *INT* The minimum depth of RNA reads required by a candidate editing site. A genomic site covered by less than this depth will be filtered [3].

--posdir *STR* The directory for genomic feature position files. The files in the directory should be named as *FeatureName*.pos (e.g. 5UTR.pos, CDS.pos, intron.pos, 3UTR.pos, ncRNA.pos, repeat.pos, etc.). If a file with the name of CDS.pos is provided, the function of inferring the codon and amino acid change after RNA editing is activated [null].

--editLevel *Float* The minimum editing level required by a candidate editing site; range from 0 to 1 [0.05].

--editDepth *INT* The minimum number of RNA reads supporting editing for a candidate editing site [3].

--extremeLevel *NUM* Exclude editing sites with extreme editing levels (100%); '1' for yes, '0' for no [0].

--refined *NUM* Whether refined the number of RNA reads supporting candidate editing sites; '1' for yes, '0' for no [1].

--refinedDepth *INT* The minimum number of RNA reads in the middle of its length supporting editing for a candidate editing site. (e.g. from positions 23-68 of a 90-bp read) [1].

--readType *INT* The minimum number of *unique* RNA reads supporting editing for a candidate editing site [3].

--junctionCoordinate *FILE* The file named junctionFlankSequenceRegion.txt created by the RES-Scanner alignment --junction option, applicable only for the input reference genome including exonic sequences surrounding splicing junctions [null].

--editPvalue *Float* The cutoff of binomial test FDR for candidate editing sites [0.05].

--ploidy *INT* Ploidy level of the samples: 1 for haploid, 2 for diploid, 3 for triploid, 4 for tetraploid, etc. [2].

--paralogous_R *NUM* Remove candidate editing sites from those regions that are similar to other parts of the genome by BLAT alignment; '1' for yes, '0' for no [1].

--paralogous_D *NUM* Discard candidate editing sites with DNA reads depth of more than twice the genome-wide peak or mean depth; '1' for yes, '0' for no [1].

--homopolymer *NUM* Remove candidate editing sites in homopolymer runs of ≥ 5 bp; '1' for yes, '0' for no [1].

--intronic *NUM* Remove intronic candidate editing sites occurring within n bases of a splice site [6].

--knownSNP *FILE* The file of known SNPs in GFF3 format [null].

--rmdup *NUM* Remove PCR duplicates for BAM file; '1' for yes, '0' for no [1].

--bestHitRatio *Float* The proportion of qualifying reads relative to all BLAT-realigned reads. Note: force --paralogous_R [0.5].

--uniqTag *NUM* Identify the unique mapping, without suboptimal hits, reads in BAM file with the tags 'XT:A:U', 'X0:i:1' and 'X1:i:0' or base on flag value; '1' for tags, '0' for flag [1]. Note: If the BAM file was generated by the RES-Scanner alignment pipeline, please set --uniqTag 1 to infer unique alignment.

--samtools *FILE* The absolute path of the SAMtools package pre-installed on the local machine (mandatory).

--blat *FILE* The absolute path of the BLAT software pre-installed on the local machine (mandatory).

--run Run the jobs in a serial working mode (i.e. run the jobs one by one automatically).

--help Show the help information.

**Parameters for homozygous genotype calling:**

--method *STR* Method for calling homozygous genotypes: Bayesian, Binomial or Frequency [Bayesian].

--HomoPrior *Float* The prior probability for a genomic position to be homozygous (force --method Bayesian) [0.99].

--rate *NUM* The rate of transitions over transversions of the genome (force --method Bayesian) [2].

--Bayesian_P *Float* The minimum Bayesian posterior probability cutoff for calling a homozygous genotype; range from 0 to 1, the bigger the better (force --method Bayesian) [0.95].

--Binomial_P *Float* The maximum p-value cutoff of the binomial test for calling a homozygous genotype; range from 0 to 1, the smaller the better (force --method Binomial) [0.05].

--Binomial_FDR *Float* The maximum FDR cutoff of the binomial test for calling a homozygous genotype; range from 0 to 1, the smaller the better (force --method Binomial) [0.05].

--Frequency_N *NUM* The maximum count of the alternative allele present in the DNA-seq data for a candidate editing site (force --method Frequency) [0].

--Frequency_R *Float* The maximum frequency of the alternative allele present in the DNA-seq data for a candidate editing site; range from 0 to 1 (force --method Frequency) [0].

Usage:

1. For strand-specific RNA-seq data:

perl RES-Scanner_identification.pl --config config.file --out./outdir/ --genome reference.fa --ss 1 --samtools /absolute_path/samtools --blat /absolute_path/blat

1. For non-strand-specific RNA-seq data:

perl RES-Scanner_identification.pl --config config.file --out ./outdir/ --genome reference.fa --ss 0 --samtools /absolute_path/samtools --blat /absolute_path/blat

Note: After running RES-Scanner_identification.pl, a shell script named RES-Scanner_identification.sh will be generated in the output directory. If option --run is not activated, users need to run this script with the command sh RES-Scanner_identification.sh, or submit the script as a job to a computing server. As the script contains four steps, for which shell scripts are also generated in the same directory, users can also run these four steps manually, one by one. It should be noted that steps 1, 2 and 3 may involve multiple parallel command lines, and we recommend that users run these command lines in parallel by submitting them as jobs to computing servers simultaneously. If option --run is activated, all of the jobs in each of the four steps will be run automatically, one by one.

**Output**

The file with the name RES_final_result.txt contains the final RNA-editing sites. If the --posdir option is invoked, the final results, with annotated genomic features, are generated in the file RES_final_result.annotation, which has the format:

- Chromosome
- Coordinate
- Strand
- Gbase: reference base
- EditType
- SampleID.DNA_baseCount[A,C,G,T]
- SampleID.RNA_baseCount[A,C,G,T]; P-value (the candidate editing sites satisfying all the criteria are marked with an asterisk *)
- TargetedGenomicFeature
- TargetedFeatureID
- CodonChange
- AminoAcidChange

**Example for test dataset**

We present a detailed example of running RES-Scanner with test data. The test data used can be downloaded from https://github.com/ZhangLabSZ/RES-Scanner. Assuming that the test data has been placed in /usr/bin/testData/, FASTQ files of both paired-end DNA-seq data and strand-specific paired-end RNA-seq data for three samples will be found in /usr/bin/testData/fastq/DNA/ and /usr/bin/testData/fastq/RNA/, respectively; the reference genome will be in /usr/bin/testData/reference/ and position files of some genomic features in /usr/bin/testData/posdir/. Before running the RES-Scanner alignment pipeline, a configuration file for the pipeline must be created manually. The configuration file is a tab-delimited table with six columns containing details of the RNA-seq and DNA-seq data representing:

- Tag ('DNA' or 'RNA')
- Sample ID
- Lane ID
- Insert size
- Filename for first FASTQ file
- Filename for second FASTQ file

The following illustrates the content of a manually created RES-Scanner_alignment_config.txt file, placed in directory /usr/bin/testData/:

DNA gynes DNA_gynes 500 /usr/bin/testData/fastq/DNA/DNA_gynes/DNA_gynes_1.fq.gz /usr/bin/testData/fastq/DNA/DNA_gynes/DNA_gynes_2.fq.gz

DNA large_workers DNA_large_workers 500 /usr/bin/testData/fastq/DNA/DNA_large_workers/DNA_large_workers_1.fq.gz /usr/bin/testData/fastq/DNA/DNA_large_workers/DNA_large_workers_2.fq.gz

DNA small_workers DNA_small_workers 500 /usr/bin/testData/fastq/DNA/DNA_small_workers/DNA_small_workers_1.fq.gz /usr/bin/testData/fastq/DNA/DNA_small_workers/DNA_small_workers_2.fq.gz

RNA gynes RNA_gyne_heads 100000 /usr/bin/testData/fastq/RNA/RNA_gyne_heads/RNA_gyne_heads_1.fq.gz /usr/bin/testData/fastq/RNA/RNA_gyne_heads/RNA_gyne_heads_2.fq.gz

RNA large_workers RNA_large_worker_heads 100000 /usr/bin/testData/fastq/RNA/RNA_large_worker_heads/RNA_large_worker_heads_1.fq.gz /usr/bin/testData/fastq/RNA/RNA_large_worker_heads/RNA_large_worker_heads_2.fq.gz

RNA small_workers RNA_small_worker_heads 100000 /usr/bin/testData/fastq/RNA/RNA_small_worker_heads/RNA_small_worker_heads_1.fq.gz /usr/bin/testData/fastq/RNA/RNA_small_worker_heads/RNA_small_worker_heads_2.fq.gz

Let us assume that the scripts of RES-Scanner have been placed in /usr/bin/RES-Scanner/, and you want to place the output files of RES-Scanner alignment in directory /usr/bin/BWA_alignment/. The absolute paths of pre-installed BWA, SAMtools and BLAT software are /usr/bin/bwa-0.5.9/bwa, /usr/bin/samtools-0.1.18/samtools and /usr/bin/blat_64/blat, respectively. Remain in the working directory /usr/bin/, and run the first part of the RES-Scanner alignment pipeline with the following command line:

perl /usr/bin/RES-Scanner/RES-Scanner_alignment/RES-Scanner_alignment.part1.pl –config /usr/bin/RES-Scanner_alignment_config.txt --outDir /usr/bin/BWA_alignment/ --ref /usr/bin/testData/reference/reference.fa --bwa /usr/bin/bwa-0.5.9/bwa --index 1

Now the shell scripts of steps zero and one will have been generated in directory /usr/bin/BWA_alignment/. These two shell scripts can now be run one after the other with the following commands. They can also be submitted as jobs to a computing server, but please ensure that all current jobs have completed successfully before proceeding to the next step:

- sh /usr/bin/BWA_alignment/step0.sh
- sh /usr/bin/BWA_alignment/step1.sh ## or run each command line in step1.sh in parallel

Part 2 of the RES-Scanner alignment pipeline can then be run with the following command line:

perl /usr/bin/RES-Scanner/RES-Scanner_alignment/RES-Scanner_alignment.part2.pl --config BWA_config.txt --outDir /usr/bin/BWA_alignment/ --ref /usr/bin/testData/reference/reference.fa --bwa /usr/bin/bwa-0.5.9/bwa --samtools /usr/bin/samtools-0.1.18/samtools --index 1

Shell scripts for steps two and three will be generated in directory /usr/bin/BWA_alignment too. Run the two shell scripts one by one with the following command lines. You can also submit them as jobs to your computing server, but again please ensure that all current jobs have completed successfully before proceeding to the subsequent step:

- sh /usr/bin/BWA_alignment/step2.sh ## or run each command line in step2.sh in parallel
- sh /usr/bin/BWA_alignment/step3.sh ## or run each command line in step3.sh in parallel

After completing the RES-Scanner alignment pipeline, users will find another configuration file, named RES-Scanner_indentification_config.txt, which contains the sample information of the BAM files generated automatically in directory /usr/bin/BWA_alignment/. The specific content of the file appears thus:

gynes /usr/bin/BWA_alignment/DNA/gynes/gynes.merge.bam /usr/bin/BWA_alignment/RNA/gynes/gynes.merge.bam

large_workers /usr/bin/BWA_alignment/DNA/large_workers/large_workers.merge.bam /usr/bin/BWA_alignment/RNA/ large_workers/large_workers.merge.bam

small_workers /usr/bin/BWA_alignment/DNA/small_workers/small_workers.merge.bam /usr/bin/BWA_alignment/RNA/small_workers/small_workers.merge.bam

For the next stage, RNA-editing site identification, the RES-Scanner identification pipeline must be run with the following command line:

perl /usr/bin/RES-Scanner/RES-Scanner.pl --config /usr/bin/BWA_alignment/RES-Scanner_indentification.pl_config.txt --out /usr/bin/RES-Scanner_identification_outdir/ --genome /usr/bin/testData/reference/reference.fa --ss 1 --samtools /usr/bin/samtools-0.1.18/samtools --blat /usr/bin/blat_64/blat --ploidy 2 --posdir /usr/bin/testData/posdir/

Four shell scripts will be generated in the output directory /usr/bin/RES-Scanner_identification_outdir/. Run the four shell scripts one after another with the following command lines. They can also be submitted as jobs to a computing server, but please ensure that all the jobs have completed successfully before proceeding to the subsequent step:

- sh /usr/bin/RES-Scanner_outdir/RES_step1.sh ## or run each command line in RES_step1.sh in parallel
- sh /usr/bin/RES-Scanner_outdir/RES_step2.sh ## or run each command line in RES_step2.sh in parallel
- sh /usr/bin/RES-Scanner_outdir/RES_step3.sh ## or run each command line in RES_step3.sh in parallel
- sh /usr/bin/RES-Scanner_outdir/RES_step4.sh

Finally, a file named RES_final_result.annotation will be generated in directory /usr/bin/RES-Scanner_identification_outdir/. This is the final result of the pipeline. If --posdir is not activated, a file named RES_final_result.txt is the result file.

**Reminder:** If you do not want to run the multiple steps in RES-Scanner alignment or RES-Scanner identification one by one manually, you can add the option --run in the main scripts, so that all the steps in each main script will be run one by one automatically. However, this means sacrificing the opportunity to run the command lines in some steps in parallel (see notes in above example), and this may greatly increase the elapsed time for the whole pipeline, especially if there is a large amount of data to process.
